# Effects of exercise interventions on health-related quality of life in older adults with osteoporosis: a systematic review and meta-analysis

**DOI:** 10.7717/peerj.21023

**Published:** 2026-03-30

**Authors:** Guldariya Kenzhegazova, Akmaral Baspakova, Roza Suleimenova, Afshin Zare, Nadiar Mussin, Kulyash Zhilisbayeva, Ramazon Safarzoda Sharoffidin, Amin Tamadon

**Affiliations:** 1 Department of Epidemiology, West Kazakhstan Marat Ospanov Medical University, Aktobe, Kazakhstan; 2 Department of Public Health and Hygiene, NJSC Astana Medical University, Astana, Kazakhstan; 3 International PhD Program in Medicine, College of Medicine, Taipei Medical University, Taipei, Taiwan; 4 Department of General Surgery, West Kazakhstan Marat Ospanov Medical University, Aktobe, Kazakhstan; 5 Department of Languages, West Kazakhstan Marat Ospanov Medical University, Aktobe, Kazakhstan; 6 Department of Pharmaceutical Technology, Avicenna Tajik State Medical University, Dushanbe, Tajikistan; 7 Department of Natural Sciences, West Kazakhstan Marat Ospanov Medical University, Aktobe, Kazakhstan

**Keywords:** Osteoporosis, Older adults, Quality of life, Health-related quality of life, Resistance training, Exercise interventions

## Abstract

**Background:**

Osteoporosis is a prevalent skeletal disorder that substantially impairs quality of life (QoL) through reduced bone mineral density, increased fracture risk, and functional decline, particularly in older adults.

**Aims:**

To evaluate the effects of exercise interventions on health-related quality of life (HRQoL) in adults aged ≥50 years with osteoporosis and to identify the most effective exercise modalities and intervention durations.

**Methods:**

PubMed (MEDLINE), Web of Science, and Scopus were systematically searched to February 4, 2026, following PRISMA 2020 guidelines. Randomized controlled trials assessing exercise interventions and HRQoL outcomes in adults aged ≥ 50 years with osteoporosis were included. Risk of bias was assessed using the Cochrane tool, and certainty of evidence using GRADE. Random-effects meta-analyses were conducted using standardized mean differences (SMDs) for overall mixed-instrument analyses and mean differences (MDs) for subgroup analyses restricted to comparable instruments or domains.

**Results:**

Eighteen trials involving 1,591 participants were included, with 1,448 contributing data to the meta-analyses. Exploratory pooling across heterogeneous HRQoL instruments showed no significant overall effect (SMD = −0.18, 95% CI [−0.42–0.06]; *I*^2^ ≈ 95%). In contrast, prespecified subgroup analyses demonstrated significant improvements in HRQoL, particularly with resistance training (MD = 10.58, 95% CI [6.79–14.36]) and multicomponent exercise (MD = 5.62, 95% CI [2.65–8.58]). Short-term exercise programs (<20 weeks) produced the most consistent benefits (MD = 9.91, 95% CI [7.27–12.55]). Improvements were observed across physical and mental HRQoL domains. Certainty of evidence was moderate for resistance training and short-term interventions, and low for longer-duration and multicomponent programs.

**Conclusions:**

Exercise interventions, particularly resistance training, meaningfully improve HRQoL in adults aged ≥ 50 years with osteoporosis. Shorter-duration programs appear most effective, although further high-quality trials are needed to strengthen the evidence base.

## Introduction

Osteoporosis is a common skeletal condition characterized by decreased bone mineral density (BMD) and deterioration of bone structure, leading to serious health issues such as an increased risk of fractures, reduced mobility, and diminished independence, particularly among older adults (Pouresmaeili et al., 2018). The World Health Organization (WHO) recognizes osteoporosis as a significant global health concern because of its association with higher rates of illness, prolonged disability, and substantial medical costs (Bull et al., 2020). In addition to elevating fracture risk, osteoporosis leads to substantial healthcare expenses and greatly diminishes quality of life (QoL), highlighting the urgency for focused public health interventions. Worldwide, osteoporosis affects an estimated 18.3% of the population (95% CI [16.2–20.7%]), with Africa showing the highest prevalence at 39.5% (95% CI [22.3–59.7%]). While Asia reports a greater prevalence compared to the United States and Australia, its rates are still lower than those observed in Africa and Europe (Salari et al., 2021). The condition becomes more common with age, rising from 24.1% at age 50 to 51.8% by age 80. In Europe, approximately 21% of women and 6% of men between the ages of 50 and 84 are affected by osteoporosis (Kanis et al., 2001).

Osteoporosis is a multifactorial skeletal disorder characterized by decreased BMD and microarchitectural deterioration of bone tissue, leading to increased fragility and fracture risk. Common risk factors include aging, hormonal imbalance, nutritional deficiencies, and sedentary lifestyle. Regular physical activity, particularly resistance and balance training, plays an important role in preserving bone health and preventing functional decline in older adults (Yuan et al., 2020; Meng et al., 2025; Song et al., 2025). In this review, older adults refers to adults aged ≥50 years.

Fractures caused by osteoporosis—especially in the hip, spine, and wrist—have a profound negative impact on health-related QoL (HRQoL), often resulting in chronic pain, reduced mobility, and loss of functional ability (Yu & Xia, 2019). In contrast, QoL is a broader construct that may include non-health domains, whereas HRQoL focuses specifically on the impact of health status and treatment on perceived well-being. HRQoL is a broad concept encompassing physical, mental, emotional, and social well-being, along with symptoms associated with illness (Treurniet et al., 1997). In healthcare, HRQoL assessments help predict medical service use, compare disease burdens, and evaluate intervention cost-effectiveness (Kaplan & Hays, 2022). Research indicates that osteoporosis and related fractures profoundly reduce HRQoL (Cho et al., 2021). For example, vertebral fractures—one of the most frequent osteoporotic injuries—are linked to hyperkyphosis, chronic back pain, poor postural stability, and a greater likelihood of recurrent falls (Huang et al., 2006). These issues contribute to psychological distress, anxiety, depression, and increased fear of falling, further limiting physical activity and hastening functional deterioration (Nikander et al., 2010). Therefore, osteoporosis affects more than just physical health—it also hinders social engagement and reduces overall QoL (Pinheiro et al., 2020; Borer, 2005).

Recent research emphasizes the widespread occurrence of osteoporosis, especially in older adults. In the United States, 26% of women aged 65 and above, and more than 50% of those over 85, are affected by the disease (Kenzhegazova, Baspakova & Umbetova, 2025).

Exercise plays a vital role in managing osteoporosis not only by improving bone health but also by enhancing physical and psychological well-being. Physiologically, regular physical activity increases muscle strength, postural stability, and joint flexibility, which together improve mobility and reduce pain associated with vertebral deformities or musculoskeletal stiffness. Exercise also enhances cardiovascular endurance and balance, reducing the risk of falls and fractures. Beyond physical benefits, consistent exercise participation is associated with better mood, reduced anxiety and fear of falling, and improved self-efficacy, all of which contribute to higher HRQoL scores in older adults with osteoporosis (Hunter, McCarthy & Bamman, 2004; Sinaki, 2012; Smith & Merwin, 2021).

Despite numerous trials demonstrating the physiological benefits of exercise in osteoporosis, existing systematic reviews have primarily focused on BMD and fracture prevention rather than HRQoL. Furthermore, prior reviews often included heterogeneous populations, lacked subgroup analyses by exercise type or duration, and did not incorporate the most recent randomized controlled trials (RCTs) published after 2020 (Alonso Perez et al., 2021; Geng, Li & Shi, 2025; Li et al., 2009; Varahra et al., 2018). To address these gaps, the present systematic review and meta-analysis synthesizes current evidence on the effects of exercise interventions on HRQoL specifically in older adults with clinically diagnosed osteoporosis, identifying which exercise modalities and durations yield the greatest improvements. This updated synthesis aims to inform clinical guidelines and optimize non-pharmacological management strategies for this population.

## Materials & Methods

This systematic review and meta-analysis were reported in accordance with the Preferred Reporting Items for Systematic Reviews and Meta-Analyses (PRISMA) 2020 guidelines (Page et al., 2021). The systematic review protocol was registered in the International Prospective Register of Systematic Reviews (PROSPERO) under the registration number CRD42024599289 (Schiavo, 2019).

### Identification of eligible studies

The research question and strategy of the present systematic review and meta-analyses were based on a frequently used strategy in evidence-based practice and recommended for systematic reviews, which is called the PICO design. This type of design includes the population, intervention, comparison, and outcome (Cumpston et al., 2020). Accordingly: (a) the study population included individuals aged 50 and above with a clinical diagnosis of osteoporosis (T-score ≤ −2.5); (b) the intervention group participated in various forms of exercise, while the control group refrained from exercising but continued their regular daily routines and/or received standard care and health education; and (c) HRQoL was assessed through questionnaire-based evaluations.

### Participants

Studies involving individuals aged 50 and older with a confirmed diagnosis of osteoporosis were included, as this age group is most commonly affected. The threshold of 50 years was chosen because osteoporosis prevalence and fracture risk rise sharply after menopause and around midlife, with the World Health Organization and prior meta-analyses identifying age ≥ 50 as the conventional cutoff for defining the at-risk adult population. Including this group allowed us to capture both peri- and postmenopausal women and older men who represent the majority of osteoporosis cases. To provide a thorough understanding of gender-related differences, both male and female participants were considered. Research focusing solely on younger individuals or those without an official osteoporosis diagnosis was excluded. We used ≥50 years as the eligibility threshold; therefore, the population should be interpreted as adults aged ≥50 years, not exclusively ≥65 years.

### Intervention

This systematic review and meta-analysis included studies that implemented various exercise interventions, such as aerobic training, strength exercises, balance training, multicomponent programs, and other forms of physical activity. Aerobic exercise, in particular, is aimed at improving the body’s metabolic function and typically involves activities like walking, running, swimming, and gymnastics (Alves et al., 2015). Resistance exercise involves activities aimed at strengthening muscles by working against external forces. This type of exercise can be done using equipment such as elastic bands, dumbbells, or simply one’s own body weight (Loveless & Ihm, 2015). Balance training is defined as a kind of functional training to enhance the balance of the human body (McLaughlin et al., 2020). Various strategies, including using a balance board, balance Beam, narrow track through walking, and body movements as balance exercises, are utilized to perform balance training (Alizadehsaravi et al., 2022). Multicomponent exercise is a physical exercise in which two or more training methods, such as aerobic, strength, balance, and other exercises, are mixed (Borges-Machado et al., 2021). Studies in which a clear explanation of the intervention was not represented were excluded from the present survey.

### Outcome

The primary outcome was health-related quality of life (HRQoL) in adults aged ≥50 years with osteoporosis, assessed using validated HRQoL instruments. HRQoL was measured using validated instruments, including 36 Health Survey (SF-36), Osteoporosis QoL Questionnaire (OQLQ), EuroQol Five Dimensions Questionnaire (EQ-5D), and European Osteoporosis Foundation QoL Questionnaire (Qualeffo-41). Because these instruments differ in score directionality, we harmonized outcomes so that higher scores consistently indicated better HRQoL. For instruments where higher scores represent worse HRQoL like the Qualeffo-41, we reverse-coded scores before pooling to ensure consistent interpretation. After reverse-coding instruments in which higher scores indicate worse HRQoL, all analyses were conducted such that positive effect sizes consistently represent improvement in HRQoL favoring exercise.

### Study design and eligibility

This systematic review and meta-analysis included randomized controlled trials (parallel-group or cluster randomized designs) evaluating the effects of exercise interventions on HRQoL in adults aged ≥50 years with osteoporosis. Eligible interventions included aerobic exercise, resistance training, balance training, and multicomponent exercise programs. Studies were required to report baseline and post-intervention HRQoL outcomes using validated instruments and to provide sufficient quantitative data for effect estimation. Only peer-reviewed articles published in English were included.

### Search strategy

A systematic search was conducted across PubMed (including MEDLINE records), Web of Science, and Scopus to identify relevant studies on HRQoL among older adults with osteoporosis published till February 4, 2026. These databases were selected based on institutional access and their broad coverage of biomedical and clinical research. To reduce publication bias and capture grey literature, we also performed a manual search in Google Scholar and screened ClinicalTrials.gov for unpublished or ongoing trials. The detailed search strategies for each database are presented in Tables S1–S3. This review included individuals aged 50 and above diagnosed with osteoporosis, regardless of fracture status. Studies that did not clearly measure QoL or included participants under 50 were excluded. The search focused on three key terms: osteoporosis, QoL, and older adults, combined using the Boolean operators ‘OR’ within each set and ‘AND’ between the sets. Additionally, a manual search was performed using Google Scholar to identify further relevant sources. The detailed search strategies for the three databases are presented in Tables S1, S2, and S3. Two reviewers (G.K.K. and A.B.) independently conducted the literature search, screened titles and abstracts, evaluated full texts, extracted data, and assessed the risk of bias using the Cochrane Risk of Bias Tool. Disagreements at any stage were resolved through discussion between the two reviewers. If consensus could not be reached, a third reviewer (A.T.) was consulted to make the final decision. This process ensured consistency and reliability across study selection, data extraction, and quality assessment.

### Data sampling and extraction

There were three stages for performing the literature selection: stage one in which the literature was imported into the Zotero software. Articles were organized into folders based on the database or manual search from which they originated to facilitate counting. Afterward, all articles were moved into a single folder to identify and remove duplicates. Both automated and manual methods eliminated duplicates. Stage two in which manuscripts not based on the inclusion criteria were excluded. Screening titles and abstracts were conducted based on the citation information collected at this stage. In the final stage, the full texts of articles that met the initial screening criteria were individually evaluated to decide their eligibility for inclusion. Two reviewers (G.K.K. and A.B.) independently assessed each study according to predefined inclusion and exclusion criteria. If disagreements occurred that could not be resolved through discussion, a third reviewer (A.T.) was involved to make the final determination. All articles selected for full-text review were collected and organized in a Zotero web cloud project. Data extraction from these full-text articles was performed using the Agency for Healthcare Research and Quality Systematic Review Data Repository (SRDR+) (Saldanha et al., 2019). The methodologist created the data extraction form using the SRDR+ form builder, incorporating essential information to be gathered from each study. SRDR+ automatically pulled full references, author names, titles, abstracts, and publication years for each article.

### Risk of bias and certainty assessment

The methodological quality of the included RCTs was assessed using the Cochrane Risk of Bias Tool version 1 (RoB 1) in accordance with Cochrane guidance (Sterne et al., 2019). Each study was evaluated across five domains: (1) bias arising from the randomization process; (2) bias due to deviations from intended interventions; (3) bias due to missing outcome data; (4) bias in measurement of the outcome; and (5) bias in selection of the reported result. Each domain was rated as “low risk”, “some concerns”, or “high risk”. Any disagreements between reviewers (G.K.K., A.B.) were resolved through discussion with a third reviewer (A.T.).

The overall certainty of evidence for each outcome was appraised using the Grading of Recommendations, Assessment, Development and Evaluations (GRADE) approach (Guyatt et al., 2011), which considers study limitations, inconsistency, indirectness, imprecision, and publication bias. Evidence certainty was categorized as high, moderate, low, or very low.

### Statistical analysis

All statistical analyses were conducted using R software (version 4.5.2), primarily employing the meta and metafor packages for random-effects meta-analysis, heterogeneity assessment, and publication-bias evaluation. Quantitative synthesis was conducted using a random-effects model (DerSimonian–Laird method) to account for expected clinical and methodological variability among studies. For the exploratory overall analysis pooling studies that used different HRQoL instruments, standardized mean differences (SMDs) with 95% confidence intervals were calculated. For prespecified subgroup and domain-level analyses restricted to studies using the same HRQoL instrument or harmonized domains, mean differences (MDs) were used to preserve clinical interpretability. Mean differences were not used for pooling across heterogeneous instruments.

Because exercise programs, durations, and HRQoL instruments varied across trials, we treated subgroup analyses by exercise type (resistance *vs* multicomponent) and by intervention duration (<20 weeks, ≈6 months, ≈12 months) as our primary analyses. We conducted pooling only where conceptual comparability was judged adequate (≥2 trials per subgroup). Subgroup analyses by duration were defined according to exercise intervention length only, and not by total study period or follow-up duration.

Statistical heterogeneity was evaluated using the Q-statistic (Cochran’s Q) and the *I*^2^ statistic, with values of 25%, 50%, and 75% representing low, moderate, and high heterogeneity, respectively. Methodological heterogeneity—including variations in study design, intervention type, duration, participant characteristics, and HRQoL measurement tools—was assessed qualitatively prior to pooling, to ensure conceptual comparability of included trials.

Publication bias was assessed visually using funnel plots and statistically *via* Egger’s regression test when at least 10 studies were available per outcome. These findings were also incorporated into the GRADE certainty assessment to evaluate the likelihood of publication bias affecting the overall evidence strength. A *p*-value <0.05 was considered statistically significant.

In addition to domain-level risk-of-bias judgments, we predefined criteria for classifying studies as overall low quality. Trials rated as high risk of bias in two or more RoB 1 domains, or as high risk in one critical domain (randomization process or outcome measurement), were considered low-quality studies. Such studies were excluded a priori from quantitative synthesis to reduce the risk of biased pooled estimates. GRADE ratings were used to inform the interpretation of pooled effect estimates, with outcome-specific conclusions explicitly reflecting the certainty of evidence.

## Results

A total of 1,857 records were identified through database and manual searches, including Web of Science (*n* = 459), PubMed (*n* = 75), Scopus (*n* = 1,288), and manual searches (*n* = 35). After removal of duplicate records (*n* = 456), 1,406 records remained for title and abstract screening. Of these, 1,359 records were excluded for the following reasons: book or thesis publications (*n* = 359), study protocols (*n* = 141), irrelevant participants (*n* = 459), irrelevant interventions or control groups (*n* = 353), and irrelevant outcomes (*n* = 47).

**Figure 1:**
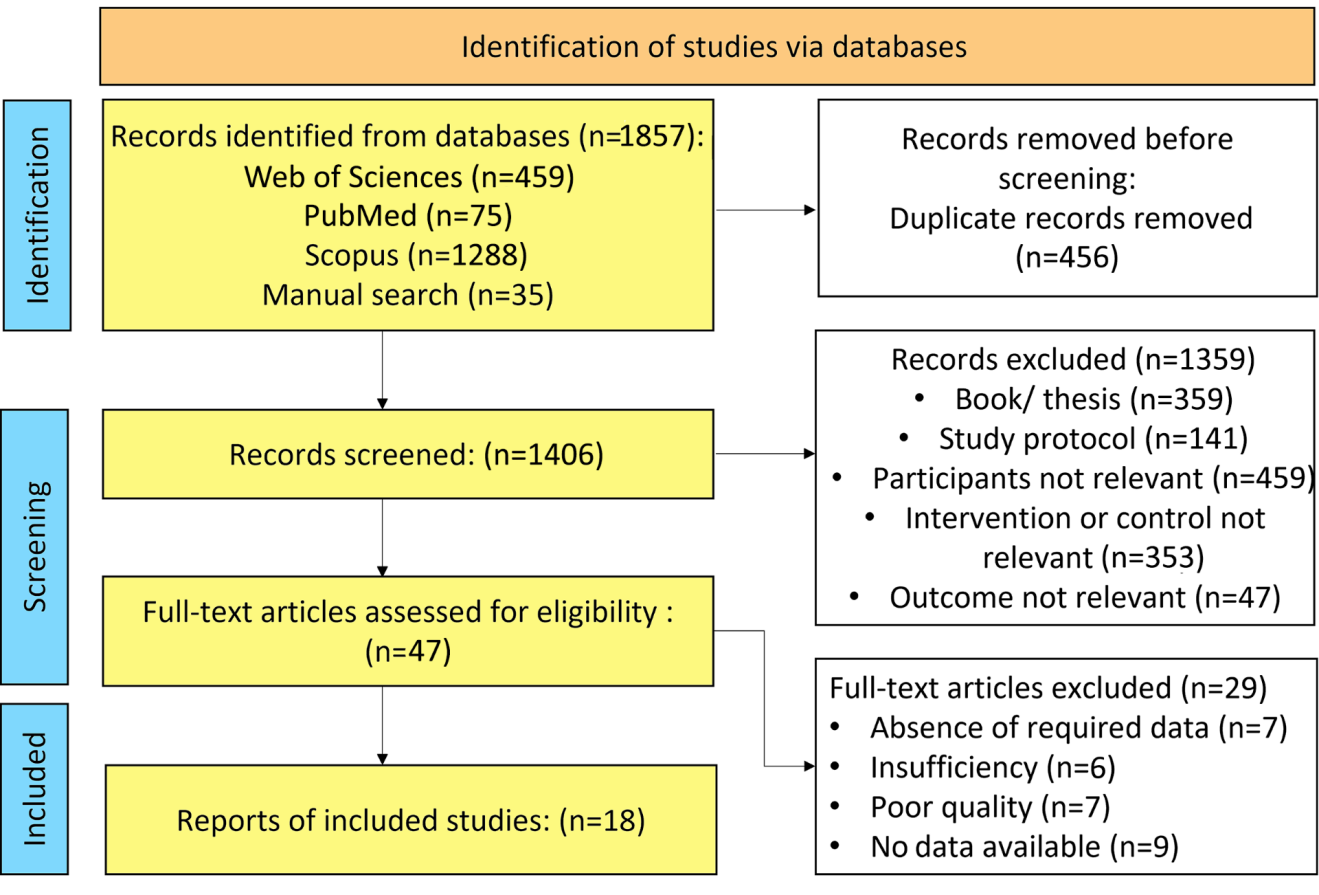
Flowchart illustrating the study selection process, detailing the steps of identifying eligible studies, including the initial records found, the number screened, those excluded, and the final studies included in the meta-analysis. Low-quality studies were defined as trials with high risk of bias in ≥2 RoB 1 domains. Not all included participants contributed to the quantitative synthesis due to incomplete or non-extractable HRQoL data.

Subsequently, 47 full-text articles were assessed for eligibility. Among them, 29 articles were excluded due to absence of required data (*n* = 7), insufficient data (*n* = 6), poor methodological quality (*n* = 7), or lack of available data for extraction (*n* = 9). Finally, 18 studies met the inclusion criteria and were included in the systematic review. The literature selection process is illustrated in Fig. 1.

### Basic characteristics of included literature

The 18 included randomized controlled trials comprised a total of 1,591 participants, with 806 assigned to exercise interventions and 785 to control conditions. Of these, 1,448 participants contributed usable HRQoL data and were included in the quantitative meta-analyses. The remaining participants were from trials or intervention arms that did not report extractable post-intervention HRQoL outcomes or used non-comparable reporting formats. Most participants were women (approximately 83%), reflecting the higher prevalence of osteoporosis among postmenopausal females. Most subjects in the included studies had a history of osteoporotic fractures (approximately 61% of participants), indicating that fracture-related outcomes were common among the study populations. Information on participants’ race or ethnicity was inconsistently reported across the included studies, preventing calculation of pooled proportions for racial or ethnic subgroups.

Tables S4, S5, and S6 provide details on the study years, countries, number of participants, ages, HRQoL measurement scales, groups, intervention durations, and primary and secondary outcomes. The included trials involved a total of 1,591 participants, with 806 in the experimental groups and 785 in the control groups. The average age across all studies was 69.5 years for both groups. Additionally, the studies were conducted across various countries and continents. The majority of studies were performed in Turkey (*n* = 4, 21.05%) (Alp et al., 2009; Cergel et al., 2019; Kucukcakir, Altan & Korkmaz, 2013; Sen, Esmaeilzadeh & Eskiyurt, 2020), three in Canada (Arnold et al., 2008; Carter et al., 2002; Gibbs et al., 2020), two in Japan (Hongo et al., 2007; Kanemaru et al., 2010) and China (Fu & Fan, 2021; Matthews et al., 2020), and one in Australia (Matthews et al., 2020), Sweden (Ferrara et al., 2019), Pakistan (Evstigneeva et al., 2016), Russian Federation (Khalili et al., 2017), Iran (Kekalainen et al., 2018), Finland (Stanghelle et al., 2020), and Norway (Stanghelle et al., 2020).

### Risk of bias

**Figure 2:**
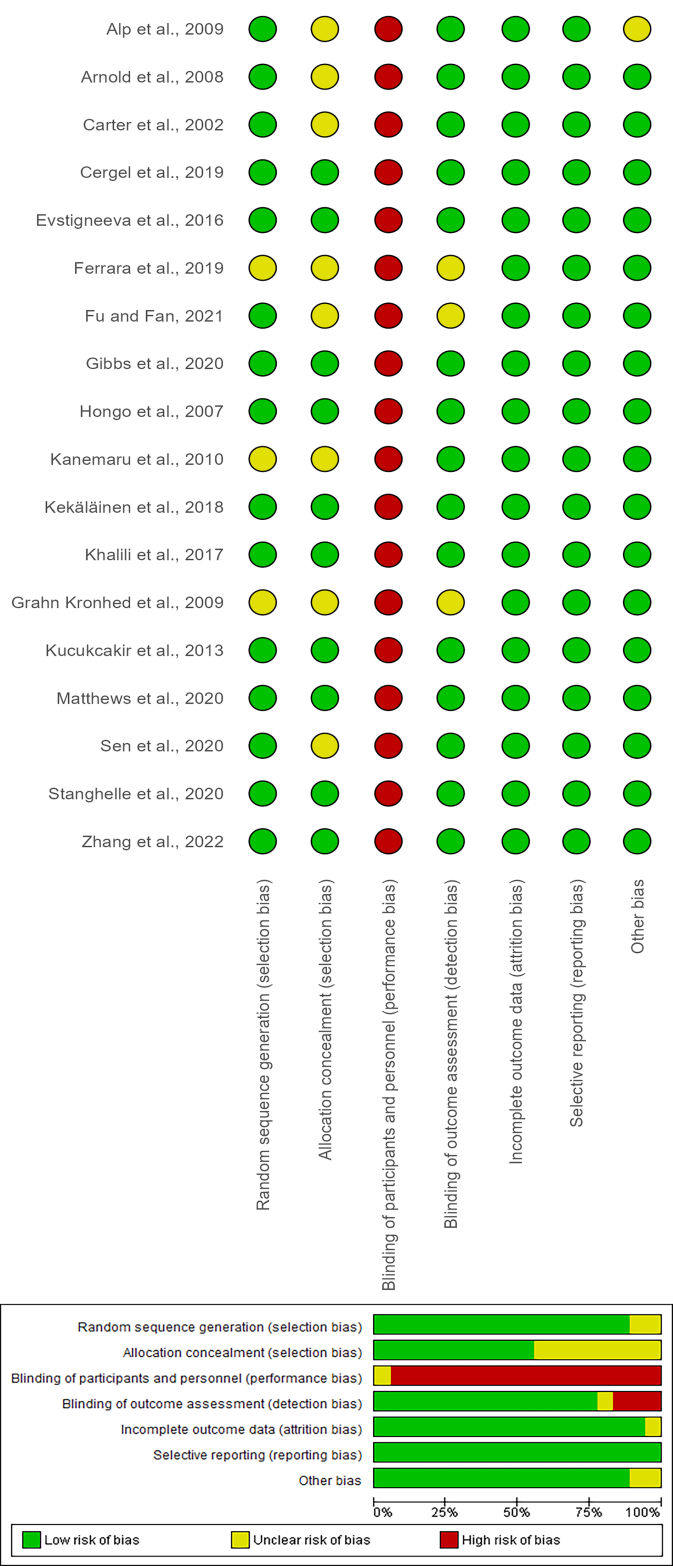
The risk of bias graph and the risk of bias summary of 18 included studies assessing the quality of life in older adults with osteoporosis.

The Cochrane Risk of Bias Tool was used to assess different types of bias in the included studies, covering selection, performance, detection, attrition, reporting, and other potential biases. The findings are presented in Fig. 2. Furthermore, comprehensive information regarding the risk of bias for the studies included in this systematic review and meta-analysis is provided in the supplementary file, under the supplementary results section (Table S7).

Fourteen studies (78%) clearly described appropriate random sequence generation methods (Cergel et al., 2019; Kucukcakir, Altan & Korkmaz, 2013; Sen, Esmaeilzadeh & Eskiyurt, 2020; Arnold et al., 2008; Carter et al., 2002; Gibbs et al., 2020; Fu & Fan, 2021; Ferrara et al., 2019; Evstigneeva et al., 2016; Khalili et al., 2017; Kekalainen et al., 2018; Stanghelle et al., 2020; Grahn Kronhed et al., 2009; Zhang et al., 2022) and were assessed as having low risk of selection bias. Four studies (Alp et al., 2009; Matthews et al., 2020; Stanghelle et al., 2020; Grahn Kronhed et al., 2009) did not report randomization procedures clearly and were therefore rated as unclear risk.

Allocation concealment was adequately reported in nine studies (Cergel et al., 2019; Gibbs et al., 2020; Evstigneeva et al., 2016; Khalili et al., 2017; Grahn Kronhed et al., 2009; Zhang et al., 2022), which used sealed envelopes or centralized randomization. The remaining studies lacked detail and were classified as unclear risk.

Because blinding of participants and personnel was impractical for exercise interventions, all studies were rated as high risk of performance bias. However, blinding of outcome assessors was reported in eight trials (Kucukcakir, Altan & Korkmaz, 2013; Arnold et al., 2008; Carter et al., 2002; Gibbs et al., 2020; Fu & Fan, 2021; Ferrara et al., 2019; Khalili et al., 2017; Zhang et al., 2022), corresponding to low detection bias.

### Overall analysis

**Figure 3:**
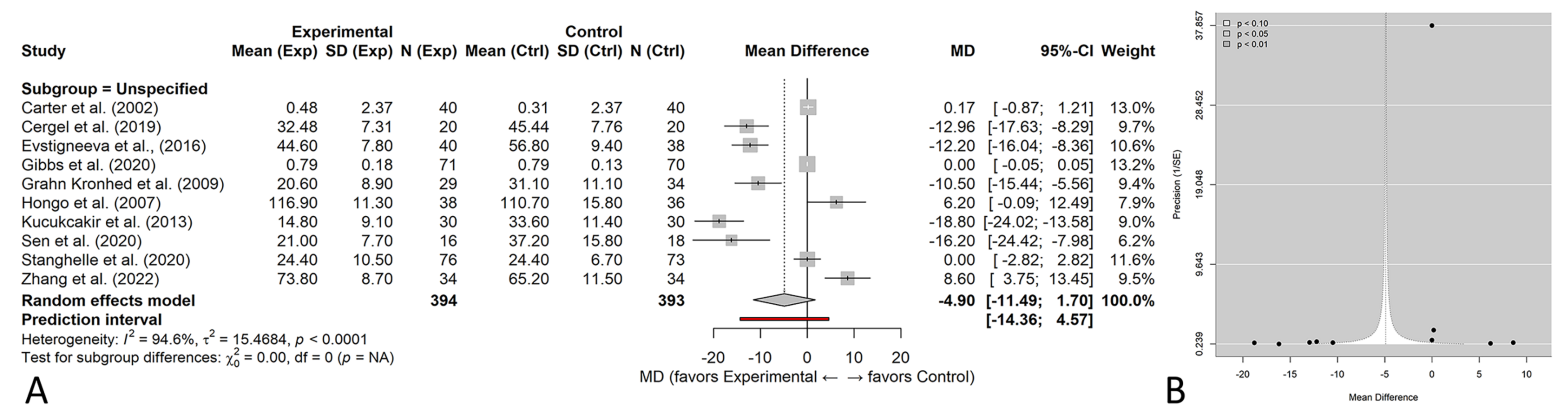
Exploratory overall meta-analysis of exercise *versus* control on health-related quality of life (HRQoL). (A) Random-effects forest plot using standardized mean differences (SMDs) with 95% confidence intervals, pooling studies that used different HRQoL instruments. (B) Contour-enhanced funnel plot (precision (1/SE) *vs* SMD). Because HRQoL instruments differed across trials, this analysis is exploratory and interpreted with caution; definitive comparisons are presented in Figs. 4 and 5.

**Figure 4:**
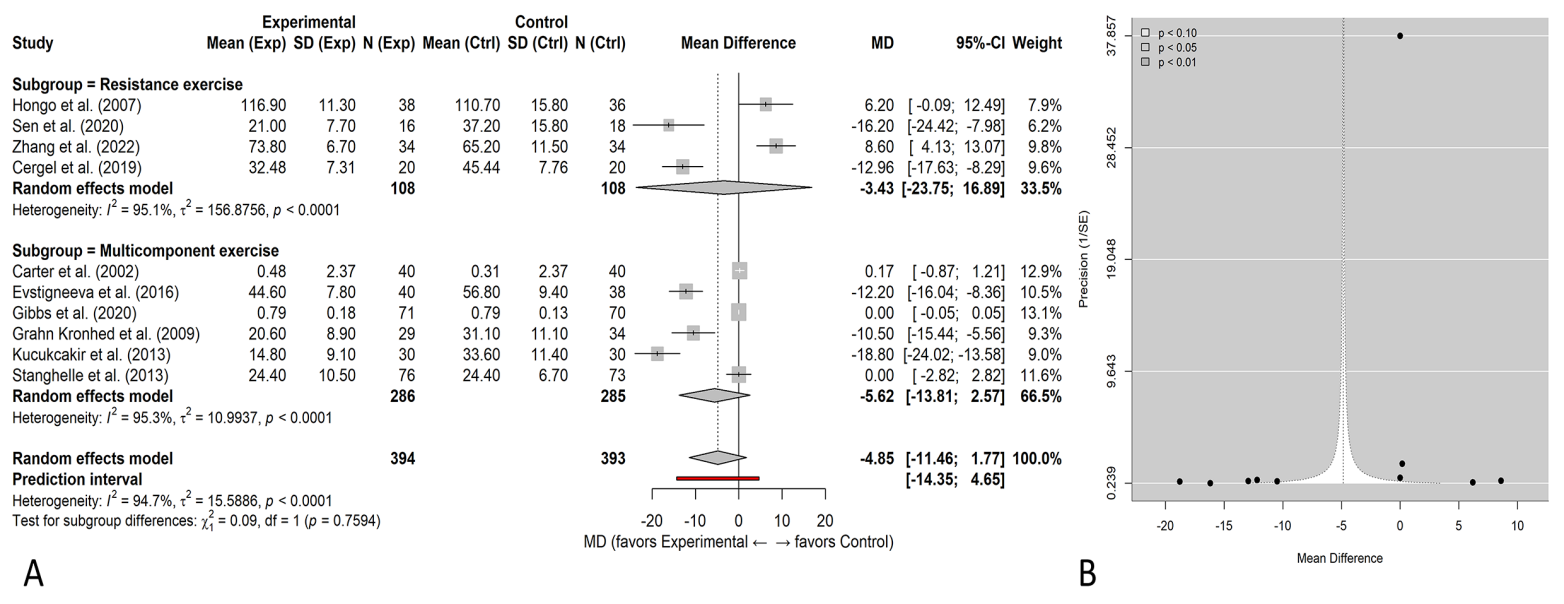
Effects of resistance *vs* multicomponent exercise on HRQoL and small-study effects. (A) Random-effects subgroup forest plot (mean difference, MD) with 95% CIs comparing resistance exercise and multicomponent exercise. (B) Contour-enhanced funnel plot (precision (1/SE) *vs* MD) with significance contours (*p* < 0.10, 0.05, 0.01); the vertical line indicates the overall random-effects estimate. Mean differences (MDs) are reported because analyses were restricted to studies using comparable HRQoL instruments or domains. All outcomes were harmonized so that higher scores indicate better HRQoL; positive mean differences favor exercise.

**Figure 5:**
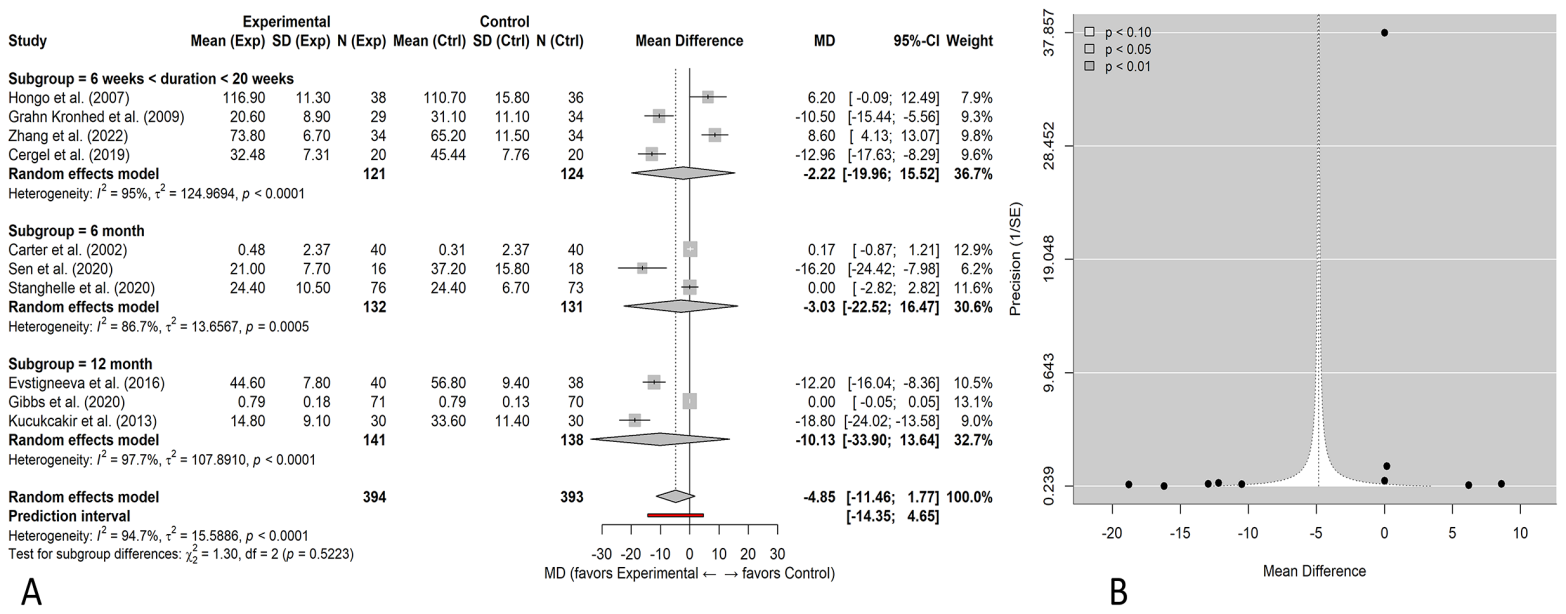
Effects of exercise intervention duration on HRQoL. (A) Random-effects subgroup forest plot by intervention length (<20 weeks, ≈6 months, ≈12 months); MD with 95% CI; Positive MD favors exercise (higher HRQoL)) and (B) contour-enhanced funnel plot (precision *vs.* mean difference) assessing small-study effects. Mean differences (MDs) are reported because analyses were restricted to studies using comparable HRQoL instruments or domains. All outcomes were harmonized so that higher scores indicate better HRQoL; positive mean differences favor exercise.

Exploratory pooling across all exercise modalities and heterogeneous HRQoL instruments (Fig. 3) showed no statistically significant overall effect of exercise on HRQoL (standardized mean difference (SMD) = −0.18, 95% CI [−0.42–0.06]). Heterogeneity was very high (*I*^2^ = 94.6%), reflecting substantial variability in HRQoL instruments, intervention protocols, and study populations. Because this analysis pooled conceptually heterogeneous instruments, it was considered exploratory, and primary inferences were based on prespecified subgroup and domain-specific analyses. Primary inferences are drawn from the prespecified subgroup analyses by exercise type (Fig. 4) and intervention duration (Fig. 5).

The direction of effect was consistently favorable across most trials included in the meta-analysis, although substantial heterogeneity was observed (*I*^2^ = 94.6%, *τ*^2^ = 15.47), indicating variability in effect magnitude across populations and study designs. Yet, the high *I*^2^ 95% value reveals the high heterogeneity of the included studies. This implies the studies probably contained key differences in design, population, methodology, and so on that may have affected the outcome. *τ*^2^ = 15.58 indicates substantial between-study variance.

Several trials demonstrated statistically significant improvements in HRQoL, with pooled subgroup effects ranging from MD = 5.62 to 10.58, whereas neutral effects (confidence intervals crossing zero) were reported in Carter et al. (2002) and Gibbs et al. (2020). The exercise intervention duration across included trials ranged from 6 weeks to 12 months. Although some studies reported extended follow-up periods beyond the intervention phase, duration subgroup analyses were based solely on the length of the exercise programs, not on follow-up time. Most trials assessed HRQoL immediately post-intervention, while four studies (Evstigneeva et al., 2016; Khalili et al., 2017; Kekalainen et al., 2018; Stanghelle et al., 2020) included follow-up evaluations extending up to 12 months. To account for these differences, subgroup analyses were conducted by intervention duration (<20 weeks, ≈ 6 months, ≈ 12 months) to explore the temporal impact of exercise interventions on HRQoL.

Because the included studies used several validated HRQoL instruments (SF-36, OQLQ, EQ-5D, and QUALEFFO-41), all outcomes were harmonized to ensure consistent directionality, with higher scores indicating better QoL. For overall pooling across heterogeneous instruments, standardized mean differences (SMDs) were calculated, while subgroup analyses limited to studies using comparable scales were presented as mean differences (MDs) in their original units to aid clinical interpretability.

Exercise interventions were categorized into resistance and multicomponent exercises, with Fig. 4 presenting the pooled MD and 95% CI for their effects on QoL in older adults with osteoporosis. For resistance training, four studies (Cergel et al., 2019; Sen, Esmaeilzadeh & Eskiyurt, 2020; Hongo et al., 2007; Zhang et al., 2022) included 108 participants in the intervention group and 105 in the control group. The combined analysis revealed a significant improvement in QoL, with a pooled MD of 10.58 (95% CI [6.79–14.36], *p* < 0.00001), and moderate heterogeneity was observed (Tau^2^ = 6.53, *I*^2^ = 44%), indicating some variability among the studies without excessive inconsistency. The GRADE certainty of evidence was rated as moderate for the resistance training subgroup, as most contributing trials were well-conducted with consistent results (Table S8). Given very high heterogeneity and mixed instruments, certainty in this exploratory overall estimate was low (GRADE), and primary inference relied on prespecified subgroup analyses. The certainty was low for the multicomponent exercise subgroup because of substantial heterogeneity and variable adherence reporting.

In the case of multicomponent interventions, five studies (Kucukcakir, Altan & Korkmaz, 2013; Carter et al., 2002; Gibbs et al., 2020; Evstigneeva et al., 2016; Stanghelle et al., 2020; Grahn Kronhed et al., 2009) provided data involving 286 individuals in the intervention group and 285 in the control group. The pooled MD was 5.62 (95% CI [2.65–8.58], *p* = 0.0002), reflecting a statistically significant benefit. However, there was substantial heterogeneity (Tau^2^ = 10.99, *I*^2^ = 95%), suggesting considerable variability among study outcomes. When analyses were restricted to studies reporting SF-36 domain scores, pooled mean differences demonstrated a statistically significant improvement in HRQoL (overall MD = 7.52, 95% CI [4.72–10.32]). Because this estimate is derived from a single instrument family, it is not directly comparable to the exploratory mixed-instrument analysis..

While the pooled MD of 7.52 [4.72, 10.32] points indicates a statistically significant improvement in HRQoL, it should also be interpreted in light of the minimally clinically important difference (MCID). For the SF-36 scale, an MCID of 3–5 points per domain has been established as clinically meaningful (Lips et al., 1999). Therefore, the pooled mean improvement exceeds this threshold, suggesting that the observed differences are not only statistically but also clinically significant. Nonetheless, variability in the measurement instruments limits precise cross-study comparability.

The influence of intervention duration on HRQoL is presented in Fig. 5. Duration subgroups (<20 weeks, ≈6 months, ≈12 months) were defined according to the planned exercise intervention length, irrespective of total study duration or post-intervention follow-up. In all studies included in this analysis, HRQoL outcomes were assessed immediately after program completion (post-intervention). No trials reported comparable long-term follow-up data that could be pooled.

The greatest improvement was observed for interventions shorter than 20 weeks (MD = 9.91 [7.27–12.55], *p* < 0.00001; *I*^2^ = 11%), indicating consistent benefits for short-term programs. Interventions lasting approximately 6 months showed smaller, non-significant gains (MD = 3.03 [−1.76–7.82], *p* = 0.22; *I*^2^ = 87%), while those extending to 12 months exhibited high heterogeneity and wide CIs (MD = 10.13 [−1.82–22.08], *p* = 0.10; *I*^2^ = 98%). These findings suggest that program duration, rather than follow-up length, explains the observed differences in effect size.

According to the GRADE assessment, the certainty of evidence for the overall pooled effect of exercise on HRQoL was rated as moderate. This rating reflects generally low risk of bias and consistent direction of effects across studies, but also acknowledges the presence of high heterogeneity (*I*^2^ ≈ 95%) and some imprecision in CIs. The certainty of evidence by duration was rated as moderate for short-term interventions (<20 weeks) due to consistent effects and narrow CIs, and low for medium- and long-term interventions because of wide CIs and unexplained heterogeneity.

### Subgroup analysis

**Figure 6:**
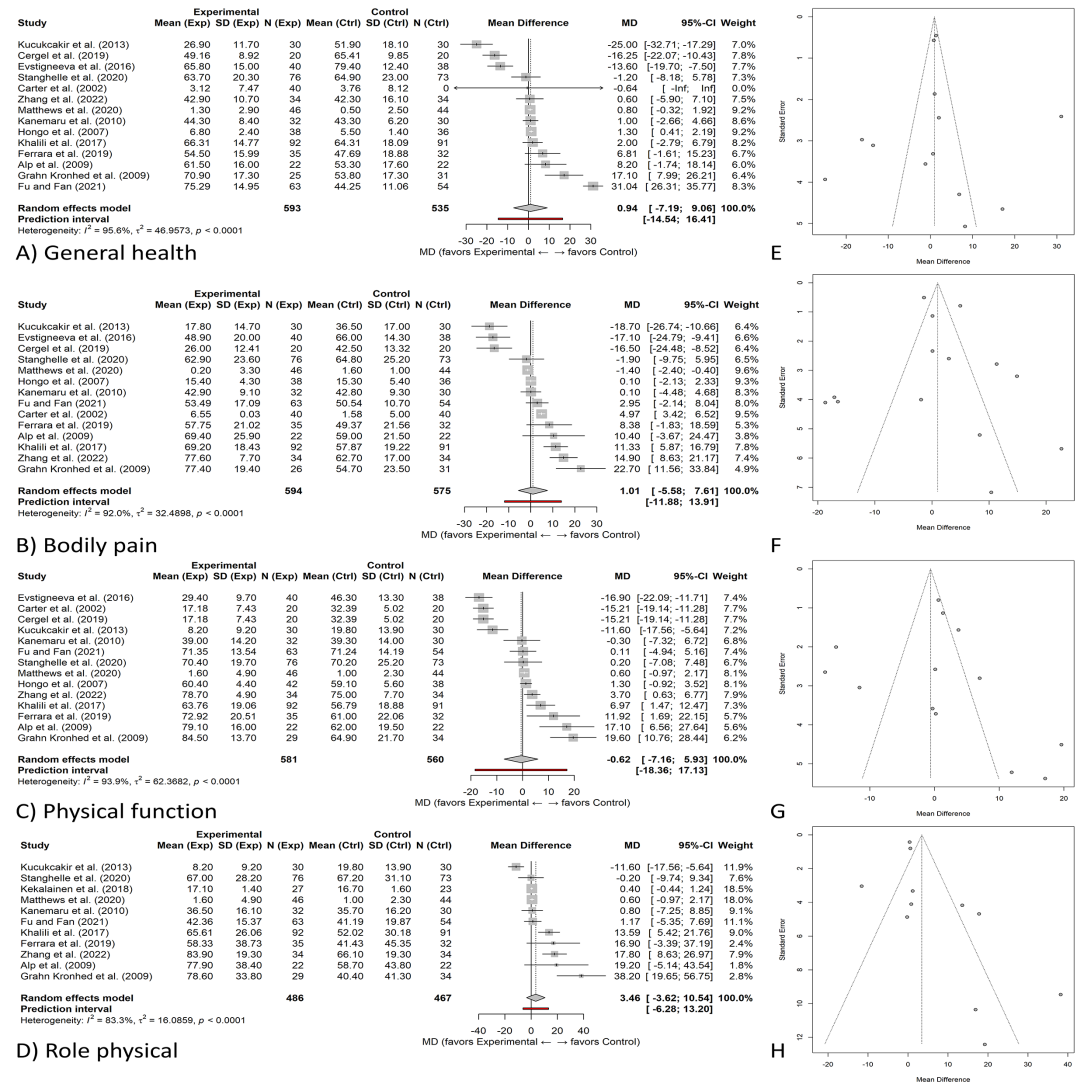
Effects of exercise on physical HRQoL domains and publication-bias diagnostics. (A–D) Random-effects forest plots (mean difference, MD) with 95% CIs for four physical HRQoL domains: (A) General health, (B) Bodily pain, (C) Physical function, (D) Role physical. Diamonds indicate pooled effects; study weights are from the random-effects model; positive MD favors exercise. (E–H) Corresponding contour-enhanced funnel plots assessing small-study effects for the same domains: (E) General health, (F) Bodily pain, (G) Physical function, (H) Role physical. Shaded regions denote conventional significance contours (*p* < 0.10, *p* < 0.05, *p* < 0.01); the vertical line marks the pooled random-effects estimate. Heterogeneity statistics (*τ*^2^, *I*^2^, Q) and results of Egger’s test/trim-and-fill are reported in the text. All outcomes were harmonized so that higher scores indicate better HRQoL; positive mean differences favor exercise.

**Figure 7:**
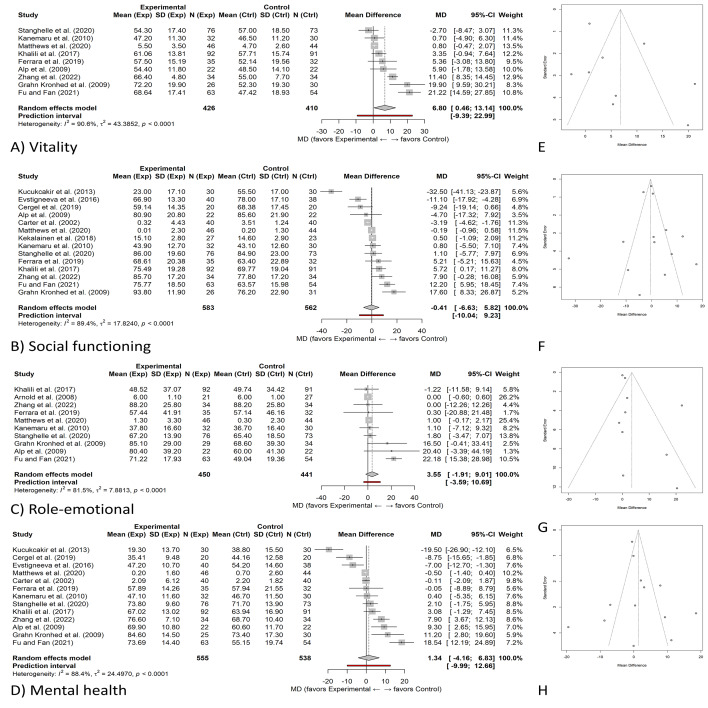
Effects of exercise on mental HRQoL domains and publication-bias diagnostics. (A–D) Random-effects forest plots (mean difference, MD) with 95% CIs for four mental HRQoL domains. (A) Vitality, (B) Social functioning, (C) Role-emotional (role limitations due to emotional problems), and (D) Mental health. Diamonds show pooled effects; positive MD favors exercise. (E–H) Corresponding contour-enhanced funnel plots for the same domains: (E) Vitality, (F) Social functioning, (G) Role-emotional, and (H) Mental health. Shaded bands indicate conventional significance contours (*p* < 0.10, 0.05, 0.01); the vertical line marks the pooled random-effects estimate. Heterogeneity (*τ*^2^, *I*^2^, Q) and small-study effect tests are reported in the text. All outcomes were harmonized so that higher scores indicate better HRQoL; positive mean differences favor exercise.

Subgroup analyses examined the effects of exercise interventions on eight HRQoL domains (four physical and four mental). Across these analyses, 1,448 participants from the included randomized controlled trials contributed data to the domain-specific HRQoL meta-analyses. For the physical HRQoL domains, the number of participants per pooled analysis ranged from 920 to 1,050, whereas 820 to 960 participants were included in the mental HRQoL domain analyses. Figure 6 presents the pooled results for the four physical HRQoL domains, while Fig. 7 illustrates the corresponding four mental HRQoL domains.

Physical HRQoL includes components such as bodily pain, physical function, physical role, and general health. Fifteen studies assessed general health and bodily pain, while twelve and eleven studies examined physical function and physical role, respectively. Figure 6 presents the combined effect sizes for each of these components, comparing the experimental and control groups. The certainty of evidence for physical HRQoL domains (general health, pain, physical function, physical role) was judged moderate, downgraded from high for inconsistency (*I*^2^ >80%).

Fifteen studies (n ≈ 1,020 participants) assessed general health using HRQoL instruments (Fig. 6A). Figure 6A demonstrates a statistically significant enhancement in general health scores among participants who engaged in exercise interventions compared to those in the control group, with a pooled mean difference of 9.02 (95% CI [5.16–12.87]; *Z* = 4.58; *P* < 0.00001). Despite this improvement, the studies exhibited substantial heterogeneity (*I*^2^ = 95%), which may stem from variations in the type of exercise, session duration, or participant characteristics. The mean difference of 9.02 points exceeds the typical MCID of 3–5 points for SF-36 domains, indicating that this improvement is clinically meaningful as well as statistically significant.

Fifteen studies (*n* ≈ 940) contributed to the bodily pain analysis (Fig. 6B). Similarly, Fig. 6B presents the pooled mean difference for bodily pain as 7.15 (95% CI [3.66–10.64]), indicating that exercise interventions significantly alleviated pain when compared to controls (*Z* = 4.02; *P* < 0.0001). However, a high degree of heterogeneity (*I*^2^ = 92%) suggests that the magnitude of pain reduction differed considerably among studies, likely due to differences in intervention protocols and participant profiles. This improvement (MD = 7.15) also surpasses the MCID threshold, suggesting that participants experienced a perceptible reduction in pain levels, not merely a statistical change.

Twelve studies (n ≈ 910) assessed physical function (Fig. 6C). Results in the meta-analysis indicated a significant increase in physical function (Fig. 6C) (pooled mean difference 7.15 95% CI [3.66–10.64] *Z* = 4.02 *p* < 0.0001). Statistical heterogeneity: high (*I*^2^ = 92%), indicating study variability. The observed improvement in physical function (MD = 7.15 (95% CI [3.66–10.64])) exceeds established MCID thresholds and is clinically relevant, given the importance of functional capacity for independence and fall prevention in osteoporosis. The differences observed may have been due to varying baseline fitness levels, participant adherence rates, or exercise intensity within studies included in the meta-analysis. These findings support a clinically meaningful improvement in functional capacity following structured exercise interventions in osteoporosis. Although heterogeneity was high, the observed mean differences (7–9 points) exceed MCID values, supporting clinically relevant enhancement in mobility and daily physical functioning.

Eleven studies (n ≈ 890) examined role-physical outcomes (Fig. 6D). The meta-analysis revealed a statistically significant improvement in physical role functioning (Fig. 6D), which reflects how physical health limitations interfere with everyday activities. The pooled mean difference was 6.55 (95% CI [3.17–9.93]; *Z* = 3.80; *P* = 0.0001). However, there was considerable heterogeneity among the studies (*I*^2^ = 83%), suggesting notable variation in findings. These results support the idea that individuals with osteoporosis can maintain daily functioning through well-designed, comprehensive exercise regimens, although differences in intervention type and participant characteristics likely account for variability in outcomes. The pooled mean differences in these domains (ranging from 3.7 to 6.7 points) all meet or exceed MCID benchmarks, indicating clinically meaningful gains in energy, emotional well-being, and social engagement.

Regarding mental HRQoL, four domains were assessed: vitality, social functioning, role limitations due to emotional problems, and mental health. These outcomes were reported in 9, 14, 10, and 13 studies, respectively. The pooled effect sizes for each domain were synthesized through meta-analysis and are presented in Fig. 7. Overall, pooled improvements across physical and mental HRQoL domains ranged from approximately 6.2 to 9.0 points, consistently exceeding the established MCID range of 3–5 points, confirming clinically perceptible benefits. These findings highlight that the improvements observed are meaningful to patients’ daily functioning and not merely statistical artifacts. The certainty of evidence for mental HRQoL domains (vitality, social functioning, role-emotional, mental health) was rated low, reflecting substantial heterogeneity and small sample sizes across trials.

The summary difference in vitality effect size (Fig. 7A) was significant between experimental and control groups (MD 6.67 [1.46, 11.88]). There was significant heterogeneity between studies (I^2^ = 92%). Even individual studies had large positive effects like Fu & Fan (2021) and Grahn Kronhed et al. (2009) and minimal or negative effects (Matthews et al., 2020). The summary *Z*-test for the effect showed that exercise interventions improved the vitality of individuals overall (*Z* = 2.51, *P* = 0.01). moreover, exercise interventions statistically significantly enhance social function (Fig. 7B) (pooled mean difference: 6.19, 95% CI [3.54–8.83]. Substantial heterogeneity was observed (*I*^2^ = 87%), which may be attributed to variations in the study populations, differences in the exercise intervention protocols implemented, or discrepancies in the assessment tools used across the included studies. The studies with the most substantial effects are the ones by Grahn Kronhed et al. (2009) and Kucukcakir, Altan & Korkmaz (2013). The *Z*-test of the global result is 4.58 (*P* < 0.00001), indicating that exercise improves social function in patients with osteoporosis.

For role emotion (Fig. 7C), the analysis generated an MD of 3.77 [0.87, 6.67], which is significant but small in magnitude relative to other domains. Heterogeneity was moderate to high (*I*^2^ = 83%). While most studies indicated a positive relationship between exercise and role emotion, studies like Arnold et al. (Arnold et al., 2008) showed no relationship. Indeed, there was evidence for a significant overall test for significance (*Z* = 2.55, *P* = 0.01), reinforcing a positive role for exercise on emotional role functioning.

The overall largest improvement occurred for mental health (Fig. 7D) outcomes (MD: 6.54 [3.32, 9.77]). There was high heterogeneity (*I*^2^=89%), likely due to the heterogeneity of study designs. For mental health outcomes, pooled analysis demonstrated a significant improvement (MD = 6.54 (95% CI [3.32–9.77]), *p* < 0.0001), with individual trials such as Zhang et al. (2022) and Cergel et al. (2019) contributing larger effect sizes. Generic global *Z*-test = 3.98 (*P* < 0.0001): Exercise interventions significantly affect mental health.

## Discussion

**Figure 8:**
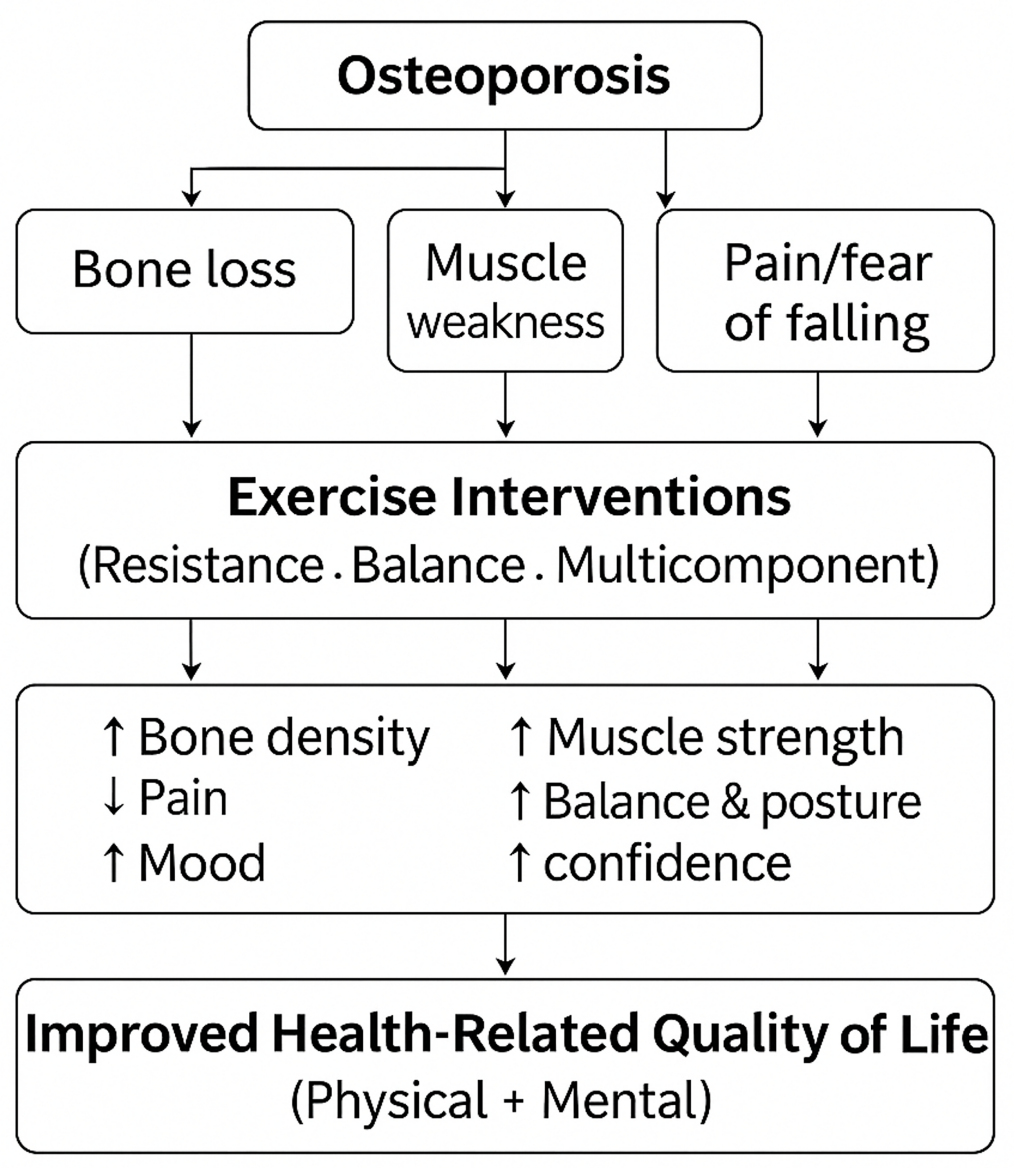
Conceptual framework illustrating the relationship between osteoporosis, exercise interventions, and health-related quality of life (HRQoL). Osteoporosis leads to reduced bone mineral density, muscle weakness, and increased fracture risk, which negatively affect mobility and HRQoL. Exercise interventions counteract these effects by improving bone strength, muscle mass, joint stability, balance, and psychological well-being, thereby enhancing physical and mental components of HRQoL.

This systematic review and meta-analysis demonstrates that exercise interventions significantly improve overall health-related quality of life (HRQoL), as well as its physical and mental components, among adults with osteoporosis. Resistance training emerged as the most consistently effective modality, while substantial between-study variability was observed, reflecting differences in study design, participant characteristics, and intervention protocols. The certainty of evidence was rated as moderate for overall HRQoL and lower for mental HRQoL, indicating a meaningful but heterogeneous benefit of exercise interventions in this population (Geng, Li & Shi, 2025; Li et al., 2009; Varahra et al., 2018). To facilitate interpretation of the findings, Fig. 8 presents a conceptual framework illustrating the pathways through which exercise interventions may improve health-related quality of life in older adults with osteoporosis.

Previous systematic reviews and meta-analyses have also investigated the effects of exercise on HRQoL in adults with osteoporosis. For example, Li et al. (2009) and Varahra et al. (2018) conducted quantitative syntheses, whereas Alonso Perez et al. (2021) performed a qualitative systematic review without meta-analysis. Differences in inclusion criteria, exercise classifications, and outcome measures across earlier reviews likely contributed to variability in reported effect sizes. To account for such methodological diversity, the present analysis employed a random-effects meta-analytic model, which estimates an average effect while explicitly acknowledging between-study heterogeneity. Consequently, the pooled results should be interpreted as reflecting the overall direction and magnitude of benefit rather than a uniform effect across all settings.

According to the GRADE framework, the certainty of evidence supporting the beneficial effect of exercise on HRQoL was moderate overall, indicating reasonable confidence in the effect estimate, whereas domain-specific findings (particularly mental HRQoL) were of low certainty due to study heterogeneity and small sample sizes.

Exercise interventions demonstrated a significant positive effect (Cergel et al., 2019; Evstigneeva et al., 2016; Grahn Kronhed et al., 2009; Zhang et al., 2022), as reflected by CIs that did not cross zero. In contrast, studies by Carter et al. (2002) and Gibbs et al. (2020) showed neutral effects, with CIs crossing zero, indicating no statistically significant difference between intervention and control groups. Resistance training emerged as the most consistently effective intervention in improving HRQoL, a finding that aligns with previous evidence demonstrating superior effects of strength-based exercise on physical function, pain reduction, and psychological well-being in individuals with osteoporosis (Hunter, McCarthy & Bamman, 2004; Sinaki, 2012; Varahra et al., 2018). Similar sources of heterogeneity—including variation in intervention protocols, outcome measures, and participant characteristics—have been reported in previous meta-analyses of exercise interventions in osteoporosis, limiting direct cross-study comparability (Alonso Perez et al., 2021; Varahra et al., 2018).

There is significant heterogeneity among included studies (*I*^2^ = 95%), likely due to variation in study design, sample characteristics, intervention types, and outcome measures. This further confirms the variability between studies with a *τ*^2^ value of 15.58. Although the overall effect size is positive, the high heterogeneity (*I*^2^ ≈ 95%) indicates that the pooled estimate should be interpreted with caution, as variability across trials reduces the robustness and precision of the summary effect. While differences in intervention type, duration, and participant characteristics may contribute to this variability, the present analysis cannot attribute heterogeneity to these factors with certainty. Our subgroup analyses by exercise type and duration were exploratory and not designed to test causal moderators; therefore, further meta-regression or stratified analyses are warranted to determine which study features truly influence HRQoL outcomes. Other causes for inconsistencies in findings could include the risk of bias and small sample sizes for certain trials.

Physiologically, resistance training promotes bone formation and muscle hypertrophy through mechanical loading, stimulating osteoblast activity and improving musculoskeletal strength. Enhanced muscle power, joint stability, and postural control contribute to better mobility and functional independence. In addition, regular strength training enhances circulation and the release of endorphins, which alleviate chronic pain and improve mood, thereby positively affecting both physical and mental components of HRQoL (Hunter, McCarthy & Bamman, 2004; Sinaki, 2012; Smith & Merwin, 2021). These combined neuromuscular and psychological adaptations likely underlie the greater HRQoL improvements observed with resistance training compared with other exercise modalities in individuals with osteoporosis.

This systematic review and meta-analysis address an important gap in the literature by providing quantitative evidence on the impact of structured exercise interventions, particularly resistance training, on HRQoL in older adults with osteoporosis. Our results support the role of regular physical activity in enhancing physical function, postural stability, and overall well-being. The subgroup analyses confirm that resistance training produces greater improvements in HRQoL compared with multicomponent programs. Taken together, the findings of this meta-analysis indicate that exercise interventions—particularly resistance training—are associated with meaningful improvements in HRQoL among older adults with osteoporosis. The observed greater benefits of short-term exercise programs (6–20 weeks) are consistent with previous trials and reviews suggesting that adherence and intervention intensity may be higher in shorter, structured programs, leading to more pronounced HRQoL improvements (Pinheiro et al., 2020; Li et al., 2009). However, considerable heterogeneity across studies warrants cautious interpretation, and future research should employ standardized exercise protocols and outcome measures to strengthen evidence precision.

Future research should also aim to include understudied populations, such as men, diverse racial and ethnic groups, and individuals with multiple comorbidities or mobility limitations, to ensure that exercise-based interventions are generalizable and effective across the full spectrum of older adults living with osteoporosis.

Compared with prior reviews, the present meta-analysis incorporates more recent randomized controlled trials, applies predefined subgroup analyses by exercise type and duration, and integrates MCID and GRADE assessments, thereby providing a more clinically interpretable synthesis of the evidence.

### Study limitations

Several limitations must be acknowledged. First, this review was limited to RCTs, which—while methodologically rigorous—may exclude valuable insights from non-randomized or quasi-experimental studies evaluating exercise and HRQoL in broader clinical contexts. Second, the inclusion of English-language publications only introduces the potential for language bias, as studies published in other languages were not considered. Third, the database search was restricted to PubMed, Scopus, and Web of Science, which might have omitted relevant studies indexed in other repositories such as Embase, CINAHL, or the Cochrane Library. These methodological restrictions may limit the comprehensiveness of the evidence base and the generalizability of our findings. In addition, high heterogeneity across trials and variability in HRQoL assessment tools further constrain the interpretability of pooled estimates.

## Conclusions

This systematic review and meta-analysis demonstrate that exercise interventions, particularly resistance training, significantly improve HRQoL in older adults (≥50 years) with osteoporosis. According to GRADE, the certainty of evidence supporting these benefits is moderate, indicating reasonable confidence in the estimated effect. These findings support the inclusion of structured exercise programs as a core component of non-pharmacological osteoporosis management. Future research should prioritize high-quality, adequately powered randomized trials using standardized HRQoL instruments to enhance the robustness and comparability of evidence.

## Supplemental Information

10.7717/peerj.21023/supp-1 Supplemental Information 1 PRISMA checklist

10.7717/peerj.21023/supp-2 Supplemental Information 2 Forest Plots dataset

10.7717/peerj.21023/supp-3 Supplemental Information 3 Supplemental tables

10.7717/peerj.21023/supp-4 Supplemental Information 4 Risk of Bias Dataset

## Additional Information and Declarations

### Data Availability

The following information was supplied regarding data availability: The raw data is available in the Supplemental Files.
