# Supplementary material for: Effects of exercise interventions on health-related quality of life in older adults with osteoporosis: a systematic review and meta-analysis
Source: PeerJ. 2026 Mar 30;14:e21023. doi: 10.7717/peerj.21023 (PMC13045839; doi:10.7717/peerj.21023)
Supplement: Supplemental Information 3 [file peerj-14-21023-s003.docx]

# Supplementary Tables

## Table S1. Scopus search strategy

| NO | Queries | Results |
| --- | --- | --- |
| #1 | TITLE-ABS-KEY ( "Osteoporosis" OR "Osteoporoses" OR "Osteoporosis, Age-Related" OR "Osteoporosis, Age Related" OR "Age-Related Osteoporosis" OR "Age-Related Osteoporoses" OR "Age Related Osteoporosis" OR "Osteoporoses, Age-Related" OR "Bone Loss, Age-Related" OR "Age-Related Bone Loss" OR "Age-Related Bone Losses" OR "Bone Loss, Age Related" OR "Bone Losses, Age-Related" OR "Osteoporosis, Senile" OR "Osteoporoses, Senile" OR "Senile Osteoporoses" OR "Senile Osteoporosis" OR "Osteoporosis, Involutional" OR "Osteoporosis, Post-Traumatic" OR "Osteoporosis, Post Traumatic" OR "Post-Traumatic Osteoporoses" OR "Post-Traumatic Osteoporosis" ) | 187,007 |
| #2 | TITLE-ABS-KEY ( "Quality of life" OR "Life Quality" OR "Health-Related Quality Of Life" OR "Health Related Quality Of Life" ) | 891,291 |
| #3 | TITLE-ABS-KEY ( "Older adults" OR "Elderly" ) | 1,214,644 |
| #4 | #1 AND #2 AND #3 and ( LIMIT-TO ( DOCTYPE , "ar" ) ) AND ( LIMIT-TO ( LANGUAGE , "English" ) ) | 1,288 |

## Table S2. PubMed search strategy.

| NO | Queries | Results |
| --- | --- | --- |
| #1 | ("Osteoporosis"[Title/Abstract] OR "Osteoporoses"[Title/Abstract] OR "Osteoporosis, Age-Related"[Title/Abstract] OR "Osteoporosis, Age Related"[Title/Abstract] OR "Age-Related Osteoporosis"[Title/Abstract] OR "Age-Related Osteoporoses"[Title/Abstract] OR "Age Related Osteoporosis"[Title/Abstract] OR "Osteoporoses, Age-Related"[Title/Abstract] OR "Bone Loss, Age-Related"[Title/Abstract] OR "Age-Related Bone Loss"[Title/Abstract] OR "Age-Related Bone Losses"[Title/Abstract] OR "Bone Loss, Age Related"[Title/Abstract] OR "Bone Losses, Age-Related"[Title/Abstract] OR "Osteoporosis, Senile"[Title/Abstract] OR "Osteoporoses, Senile"[Title/Abstract] OR "Senile Osteoporoses"[Title/Abstract] OR "Senile Osteoporosis"[Title/Abstract] OR "Osteoporosis, Involutional"[Title/Abstract] OR "Osteoporosis, Post-Traumatic"[Title/Abstract] OR "Osteoporosis, Post Traumatic"[Title/Abstract] OR "Post-Traumatic Osteoporoses"[Title/Abstract] OR "Post-Traumatic Osteoporosis"[Title/Abstract]) OR ("Osteoporosis" OR "Osteoporoses" OR "Osteoporosis, Age-Related" OR "Osteoporosis, Age Related" OR "Age-Related Osteoporosis" OR "Age-Related Osteoporoses" OR "Age Related Osteoporosis" OR "Osteoporoses, Age-Related" OR "Bone Loss, Age-Related" OR "Age-Related Bone Loss" OR "Age-Related Bone Losses" OR "Bone Loss, Age Related" OR "Bone Losses, Age-Related" OR "Osteoporosis, Senile" OR "Osteoporoses, Senile" OR "Senile Osteoporoses" OR "Senile Osteoporosis" OR "Osteoporosis, Involutional" OR "Osteoporosis, Post-Traumatic" OR "Osteoporosis, Post Traumatic" OR "Post-Traumatic Osteoporoses" OR "Post-Traumatic Osteoporosis"[MeSH Terms]) | [120,295](https://pubmed.ncbi.nlm.nih.gov/?term=%28%28Quality+of+life%5BTitle%5D%29+OR+%28Quality+of+life%5BTitle%2FAbstract%5D%29%29+OR+%28Quality+of+life%5BMeSH+Terms%5D%29&sort=) |
| #2 | ("Quality of life"[Title/Abstract] OR "Life Quality"[Title/Abstract] OR "Health-Related Quality Of Life"[Title/Abstract] OR "Health Related Quality Of Life"[Title/Abstract]) OR ("Quality of life" OR "Life Quality" OR "Health-Related Quality Of Life" OR "Health Related Quality Of Life"[MeSH Terms]) | [553,933](https://pubmed.ncbi.nlm.nih.gov/?term=%22Quality+of+life%22+OR+%22Life+Quality%22+OR+%22Health-Related+Quality+Of+Life%22+OR+%22Health+Related+Quality+Of+Life%22&sort=&filter=pubt.clinicaltrial&filter=pubt.randomizedcontrolledtrial) |
| #3 | ("Older adults"[Title/Abstract] OR "Elderly"[Title/Abstract]) OR ("Older adults" OR "Elderly"))[MeSH Terms]) | [492,014](https://pubmed.ncbi.nlm.nih.gov/?term=%22Older+adults%22+OR+%22Elderly%22&sort=&filter=pubt.clinicaltrial&filter=pubt.randomizedcontrolledtrial) |
| #4 | #1 AND #2 AND #3 | [883](https://pubmed.ncbi.nlm.nih.gov/?term=%28%28%22Osteoporosis%22+OR+%22Osteoporoses%22+OR+%22Osteoporosis%2C+Age-Related%22+OR+%22Osteoporosis%2C+Age+Related%22+OR+%22Age-Related+Osteoporosis%22+OR+%22Age-Related+Osteoporoses%22+OR+%22Age+Related+Osteoporosis%22+OR+%22Osteoporoses%2C+Age-Related%22+OR+%22Bone+Loss%2C+Age-Related%22+OR+%22Age-Related+Bone+Loss%22+OR+%22Age-Related+Bone+Losses%22+OR+%22Bone+Loss%2C+Age+Related%22+OR+%22Bone+Losses%2C+Age-Related%22+OR+%22Osteoporosis%2C+Senile%22+OR+%22Osteoporoses%2C+Senile%22+OR+%22Senile+Osteoporoses%22+OR+%22Senile+Osteoporosis%22+OR+%22Osteoporosis%2C+Involutional%22+OR+%22Osteoporosis%2C+Post-Traumatic%22+OR+%22Osteoporosis%2C+Post+Traumatic%22+OR+%22Post-Traumatic+Osteoporoses%22+OR+%22Post-Traumatic+Osteoporosis%22+AND+%28clinicaltrial%5BFilter%5D+OR+randomizedcontrolledtrial%5BFilter%5D%29%29+AND+%28%22Quality+of+life%22+OR+%22Life+Quality%22+OR+%22Health-Related+Quality+Of+Life%22+OR+%22Health+Related+Quality+Of+Life%22+AND+%28clinicaltrial%5BFilter%5D+OR+randomizedcontrolledtrial%5BFilter%5D%29%29%29+AND+%28%22Older+adults%22+OR+%22Elderly%22+AND+%28clinicaltrial%5BFilter%5D+OR+randomizedcontrolledtrial%5BFilter%5D%29%29&sort=&filter=pubt.clinicaltrial&filter=pubt.randomizedcontrolledtrial) |
| Filter | Adaptive Clinical Trial, Clinical Study, Clinical Trial, Clinical Trial, Phase I, Clinical Trial, Phase II, Clinical Trial, Phase III, Clinical Trial, Phase IV, Controlled Clinical Trial, Observational Study, Randomized Controlled Trial, English | 75 |

## Table S3. Web of Science search strategy

| NO | Queries | Results |
| --- | --- | --- |
| #1 | ((TI=(“Osteoporosis” OR “Osteoporoses” OR “Osteoporosis, Age-Related” OR “Osteoporosis, Age Related” OR “Age-Related Osteoporosis” OR “Age-Related Osteoporoses” OR “Age Related Osteoporosis” OR “Osteoporoses, Age-Related” OR “Bone Loss, Age-Related” OR “Age-Related Bone Loss” OR “Age-Related Bone Losses” OR “Bone Loss, Age Related” OR “Bone Losses, Age-Related” OR “Osteoporosis, Senile” OR “Osteoporoses, Senile” OR “Senile Osteoporoses” OR “Senile Osteoporosis” OR “Osteoporosis, Involutional” OR “Osteoporosis, Post-Traumatic” OR “Osteoporosis, Post Traumatic” OR “Post-Traumatic Osteoporoses” OR “Post-Traumatic Osteoporosis”)) OR AB=(“Osteoporosis” OR “Osteoporoses” OR “Osteoporosis, Age-Related” OR “Osteoporosis, Age Related” OR “Age-Related Osteoporosis” OR “Age-Related Osteoporoses” OR “Age Related Osteoporosis” OR “Osteoporoses, Age-Related” OR “Bone Loss, Age-Related” OR “Age-Related Bone Loss” OR “Age-Related Bone Losses” OR “Bone Loss, Age Related” OR “Bone Losses, Age-Related” OR “Osteoporosis, Senile” OR “Osteoporoses, Senile” OR “Senile Osteoporoses” OR “Senile Osteoporosis” OR “Osteoporosis, Involutional” OR “Osteoporosis, Post-Traumatic” OR “Osteoporosis, Post Traumatic” OR “Post-Traumatic Osteoporoses” OR “Post-Traumatic Osteoporosis”)) OR AK=(“Osteoporosis” OR “Osteoporoses” OR “Osteoporosis, Age-Related” OR “Osteoporosis, Age Related” OR “Age-Related Osteoporosis” OR “Age-Related Osteoporoses” OR “Age Related Osteoporosis” OR “Osteoporoses, Age-Related” OR “Bone Loss, Age-Related” OR “Age-Related Bone Loss” OR “Age-Related Bone Losses” OR “Bone Loss, Age Related” OR “Bone Losses, Age-Related” OR “Osteoporosis, Senile” OR “Osteoporoses, Senile” OR “Senile Osteoporoses” OR “Senile Osteoporosis” OR “Osteoporosis, Involutional” OR “Osteoporosis, Post-Traumatic” OR “Osteoporosis, Post Traumatic” OR “Post-Traumatic Osteoporoses” OR “Post-Traumatic Osteoporosis”) | 106,104 |
| #2 | ((TI=(“Quality of life” OR “Life Quality” OR “Health-Related Quality Of Life” OR “Health Related Quality Of Life”)) OR AB=(“Quality of life” OR “Life Quality” OR “Health-Related Quality Of Life” OR “Health Related Quality Of Life”)) OR AK=(“Quality of life” OR “Life Quality” OR “Health-Related Quality Of Life” OR “Health Related Quality Of Life”) | 510,528 |
| #3 | ((TI=(“Older adults” OR “Elderly”)) OR AB=(“Older adults” OR “Elderly”)) OR AK=(“Older adults” OR “Elderly”) | 519,123 |
| #4 | #1 AND #2 AND #3 | 692 |
| Filter | Document Types: Articles; languages: English | 459 |

## Table S4. Study characteristics of surveys in which the quality of Life of older people with osteoporosis are evaluated

| Author, year (reference) | Study Design | Country | Setting | Exercise intervention duration |
| --- | --- | --- | --- | --- |
| Alp et al., 2009 (1) | Prospective, randomized, longitudinal, parallel, cohort, and single-blinded study | Turkey | Uludağ University Hospital and Atatürk Balneotherapy and Rehabilitation Center | 6 months |
| Arnold et al., 2008 (2) | - | Canada | University of Saskatchewan and Saskatoon Osteoporosis Centre in Saskatchewan | 20 weeks |
| Carter et al., 2002 (3) | Prospective, Randomized, Longitudinal, Parallel, Cohort, Blinded, Single-blinded study | Canada | Community centers | 20 weeks |
| Cergel et al., 2019 (4) | Prospective, Randomized, Longitudinal, Parallel, Cohort, Open-label study | Turkey | Department of Physical Medicine and Rehabilitation at Pamukkale University | 6 weeks |
| Evstigneeva et al., 2016 (5) | Prospective, Randomized, Longitudinal, Parallel, Case-control, Blinded, Single-blinded study | Russia | Sverdlovsk Regional Clinical Hospital No. 1 and the Ural Institute of Traumatology and Orthopedics | 12 months |
| Ferrara et al., 2019 (6) | Prospective, randomized, longitudinal, parallel, cohort, and open-label study | Italy | Outpatients University Physical Medicine and Rehabilitation Hospital | Three years |
| Fu and Fan, 2021 (7) | Prospective, randomized, longitudinal, parallel, cohort, and open-label study | China | Nanhua Hospital | Three years |
| Gibbs et al., 2020 (8) | Prospective, Randomized, Longitudinal, Parallel, Cohort, Blinded, Single-blinded study | Canada and Australia | Five Canadian and two Australian academic or community | 12 months |
| Hongo et al., 2007 (9) | Prospective, randomized, longitudinal, parallel, cohort, open-label study | Japan | Akita University Hospital and Joto Orthopedic Hospital | 4 months |
| Kanemaru et al., 2010 (10) | Prospective, Randomized, Longitudinal, Parallel, Case-control, Open-label study | Japan | Tokyo Metropolitan Geriatric Hospital | 12 months |
| Kekalainen et al., 2018 (11) | Prospective, randomized, longitudinal, parallel, cohort, open-label study | Finland | University of Jyväskylä | 9 months |
| Khalili et al., 2017 (12) | Prospective, Randomized, Longitudinal, Parallel, Cohort, Blinded, Single-blinded study | Iran | Two general educational hospitals in Tehran | 6 months |
| Grahn Kronhed et al., 2009 (13) | Prospective, randomized, longitudinal, parallel, cohort, and open-label study | Sweden | Osteoporosis Unit at Linköping University Hospital | 4 months |
| Kucukcakir et al., 2013 (14) | Prospective, randomized, longitudinal, parallel, cohort, and single-blinded study | Turkey | Uludağ University Medical Faculty, Physical Medicine and Rehabilitation | One year |
| Matthews et al., 2020 (15) | Prospective, randomized, longitudinal, parallel, cohort, and open-label study | Australia | Community-dwelling men from the Geelong and surrounding areas in Victoria | Three years |
| Sen et al., 2020 (16) | Prospective, Randomized, Longitudinal, Parallel, Cohort, Blinded, Single-blinded study | Turkey | Department of Physical Medicine and Rehabilitation, Istanbul Faculty of Medicine, Istanbul University | 6 months |
| Stanghelle et al., 2020 (17) | Prospective, Randomized, Longitudinal, Parallel, Cohort, Blinded, Single-blinded study | Norway | OsloMet – Oslo Metropolitan University and a physiotherapy clinic in the Oslo area | Three years |
| Zhang et al., 2022 (18) | Prospective, Randomized, Longitudinal, Parallel, Cohort, Open-label study | China | Longhua Hospital Shanghai University of Traditional Chinese Medicine in Shanghai | 7 months |

Exercise intervention duration refers to the planned length of the exercise program used in the meta-analyses. Follow-up duration, when reported, reflects post-intervention observation and was not used to define duration subgroups.

## Table S5. Participant characteristics of surveys in which the quality of Life of older people with osteoporosis are evaluated

| Author, year (reference) | Characteristics of Participants | | | Sample Size (Participants) | | |
| --- | --- | --- | --- | --- | --- | --- |
|  | Condition | Sex | Age (year) | Total | Intervention | Control |
| Alp et al., 2009 (1) | Participants with senile osteoporosis | Female | 65 - 77 | 44 | 22 | 22 |
| Arnold et al., 2008 (2) | Participants with osteoporosis | Female | 60 years or older | 68 | 41 | 27 |
| Carter et al., 2002 (3) | Participants with osteoporosis | Female | 65 - 75 | 93 | 45 | 48 |
| Cergel et al., 2019 (4) | The participants had osteoporosis with vertebral fractures. | Female | 60.3 ± 9.3 | 60 | 40 | 20 |
| Evstigneeva et al., 2016 (5) | The participants had radiographically confirmed vertebral fractures | Female | ≥65 years | 141 | 71 | 70 |
| Ferrara et al., 2019 (6) | The participants had osteoporotic vertebral fractures and chronic back pain | Female | 69.2 ± 7.7 | 78 | 40 | 38 |
| Fu and Fan, 2021 (7) | The participants were postmenopausal women with osteoporosis | Female | 70.6 ± 8.2 | 98 | 56 | 42 |
| Gibbs et al., 2020 (8) | The participants in this study were older women with vertebral fractures | Both | CG:  62.04 ± 5.33  SG:  63.73 ± 5.64 | 117 | 63 | 54 |
| Hongo et al., 2007 (9) | The participants were postmenopausal women with osteoporosis | Female | 67 ± 7 | 80 | 42 | 38 |
| Kanemaru et al., 2010 (10) | The participants were elderly women with osteoporosis, specifically those who had vertebral fractures. | Female | Over 60 years | 69 | 37 | 32 |
| Kekalainen et al., 2018 (11) | The participants were older adults (aged 65–75) in generally good health, without any serious cardiovascular disease or other conditions that would impede participation in resistance training. | Both | 65–75 | 106 | 81 | 25 |
| Khalili et al., 2017 (12) | The participants had primary osteoporosis and kyphosis | Female | 55 - 75 | 183 | 92 | 91 |
| Grahn Kronhed et al., 2009 (13) | Participants with osteoporosis | Female | 60 - 81 | 73 | 37 | 36 |
| Kucukcakir et al., 2013 (14) | Postmenopausal participants with osteoporosis | Female | 45 - 65 | 67 | 35 | 32 |
| Matthews et al., 2020 (15) | Participants in this study are healthy community-dwelling men aged 50–79 years, with normal to below average BMD | Male | 50 - 79 | 180 | 136 | 44 |
| Sen et al., 2020 (16) | The participants were postmenopausal women with low BMD, ranging from T-scores between -2.0 and -3.0. | Female | 40 - 65 | 58 | 38 | 20 |
| Stanghelle et al., 2020 (17) | Participants with osteoporosis and a history of vertebral fractures | Female | 65 years or older | 149 | 76 | 73 |
| Zhang et al., 2022 (18) | Participants with osteoporosis | Both | 68.43 ± 4.68 | 72 | 36 | 36 |

BMD, bone mineral density. Total sample size across included trials was N = 1,591; however, only participants from studies reporting extractable HRQoL outcomes were included in the meta-analyses (N = 1,448).

## Table S6. Group and outcome(s) characteristics of surveys in which the quality of Life of older people with osteoporosis are evaluated

| Author, year (reference) | Groups | | HRQOL scale | Primary and Secondary Outcomes | | | |
| --- | --- | --- | --- | --- | --- | --- | --- |
|  | Intervention group | Control group |  | Primary | Measuring scale | secondary | Measuring scale |
| Alp et al., 2009 (1) | Practiced a Tai Chi exercise program | Received home-based active range of motion exercises but did not engage in any other physical activity or structured exercise program | + NHP  + SF-36 | Functional ability | + NHP  + SF-36 | Balance | + SRT  + TSS |
|  |  |  |  | QoL |  | Postural control |  |
| Arnold et al., 2008 (2) | Participants performed exercises in water | Participants did not engage in any exercise and were on a wait-list | OQLQ | Balance | + Berg Balance Scale  + Functional Reach Test  + Backward Tandem Walk | Functional status | + OFDQ  + OQLQ  + Chair stands  + Self-paced walking velocity |
|  | Participants performed exercises in land |  |  |  |  | QoL |  |
| Carter et al., 2002 (3) | Participants who underwent the exercise program | Participants who continued their routine daily activities | Osteoporosis-specific quality-of-life questionnaire | Dynamic balance | Figure-eight velocity | Static balance | Equitest computerized posturography platform |
|  |  |  |  | Knee extension strength | Dynamometer | HRQOL | Osteoporosis-specific quality-of-life questionnaire |
| Cergel et al., 2019 (4) | Participants who performed the back extensor strengthening exercises under supervision in a clinic | Participants who continued their daily life activities without any additional exercise or therapy | QUALEFFO-41 | Spinal pain | VAS | Back extensor muscle strength | Hand-held dynamometer |
|  | Participants who performed the exercises at home based on an instructional booklet |  |  |  |  | Trunk and arm endurance | TLS test |
|  |  |  |  |  |  | Kyphosis | Digital inclinometer |
|  |  |  |  |  |  | Functional mobility | TUGT |
|  |  |  |  |  |  | HRQOL | QUALEFFO-41 questionnaire |
| Evstigneeva et al., 2016 (5) | Participants who underwent a 12-month physical exercise program | Participants who continued their usual daily activities with no additional exercise intervention | QUALEFFO-41 | QoL | QUALEFFO-41 | Balance | Balance Master® System NeuroCom® |
|  |  |  |  |  |  | Functional mobility | TUGT and Sit-to-Stand test |
|  |  |  |  |  |  | Thoracic kyphosis | Occiput-to-wall distance |
|  |  |  |  |  |  | Incidence of fractures | - |
| Ferrara et al., 2019 (6) | Tai chi group | Usual care group | SF-36 | QoL | SF-36 | Static balance | Stabilometric-standardized exam |
| Fu and Fan, 2021 (7) | Exercise rehabilitation therapy in addition to routine treatment | Received routine treatment only, without exercise rehabilitation therapy | SF-36 | HRQOL | SF-36 | BMD | DXA |
|  |  |  |  |  |  | Blood glucose levels | Standard blood glucose tests |
|  |  |  |  |  |  | Pain | VAS |
|  |  |  |  |  |  | Adverse reactions | - |
| Gibbs et al., 2020 (8) | Home exercise group | Equal-attention control group | + EQ-5D-3L questionnaire  + mini-OQLQ | Functional performance | SPPB and BOOMER | Pain | VAS |
|  |  |  |  | Posture | Occiput-to-wall distance and standing height. | Fear of falling | FES-I |
|  |  |  |  | Patient-reported outcomes | - | Exercise self-efficacy | Exercise Self-Efficacy Questionnaire |
|  |  |  |  |  |  | Adherence to exercise | - |
| Hongo et al., 2007 (9) | This group performed low-intensity back-strengthening exercise | This group did not participate in any exercise and continued with their usual activities | JOQOL | Back extensor strength | Isometric dynamometer | Spinal range of motion | Spinal Mouse®, a hand-held electromechanical device |
|  |  |  |  |  |  | HRQOL | JOQOL |
| Kanemaru et al., 2010 (10) | Home-based muscle training | No intervention | SF-36 | Muscle strength of the upper and lower extremities | Hand dynamometer and leg strength | Walking speed | Time to walk 10 meters |
|  |  |  |  | Physical functioning | SF-36 | Grip strength | Hand dynamometer. |
|  |  |  |  | QOL | SF-36 | Dynamic balance | TUGT |
|  |  |  |  |  |  | Static balance | - |
|  |  |  |  |  |  | Total steps walked per day. | Pedometer |
| Kekalainen et al., 2018 (11) | RT1: Resistance training once a week | Non-training control group | WHOQOL-BREF questionnaire | QoL | WHOQOL-BREF questionnaire | - | - |
|  | RT2: Resistance training twice a week |  |  | SoC | Antonovsky’sSOC-13 |  |  |
|  | RT3: Resistance training three times a week |  |  | DS | BDI-II |  |  |
| Khalili et al., 2017 (12) | Participants performed back extensor strengthening exercises at home along with pharmacotherapy, weight-bearing, and balance-training exercises. | Participants received pharmacotherapy, weight-bearing, and balance-training exercises but without the back extensor strengthening exercises. | SF-36 | QOL | SF-36 | Back extensor strength | - |
|  |  |  |  |  |  | Postural stability | - |
| Grahn Kronhed et al., 2009 (13) | Supervised group training program | Continue with their usual care | + SF-36  + Qualeffo-41 | HRQOL | SF-36 | Pain | VAS |
|  |  |  |  |  | Qualeffo-41 | Balance | - |
| Kucukcakir et al., 2013 (14) | This group underwent a supervised Pilates exercise program twice a week for one year | This group performed a home exercise program consisting of thoracic extension exercises | + SF-36  + Qualeffo-41 | Pain | VAS | The number of falls experienced by participants during the one-year study period | - |
|  |  |  |  | Functional status | Six-minute walking test and the sit-to-stand test |  |  |
|  |  |  |  | HRQOL | SF-36 and Qualeffo-41 |  |  |
| Matthews et al., 2020 (15) | Exercise + fortified milk: Participants in this group received both the multicomponent exercise program and fortified milk. | Control: Participants in this group received the usual care without any exercise or fortified milk | SF-36 | HRQOL | SF-36 | - | - |
|  | Exercise alone: Participants in this group received only the multicomponent exercise program. |  |  | DS | CES-D |  |  |
|  | Fortified milk alone: Participants in this group consumed calcium–vitamin D3 fortified milk. |  |  |  |  |  |  |
| Sen et al., 2020 (16) | WBV group | Did not undergo any specific exercise intervention | QUALEFFO | BMD | DXA | Serum OC | IMMULITE 2000 system |
|  | High-impact exercise group |  |  |  |  | Serum CTX | Modular E170 analyzer |
| Stanghelle et al., 2020 (17) | Participants who underwent a 12-week supervised multicomponent resistance and balance exercise program | Participants who received usual care and were instructed to maintain their normal activities | + SF-36  + Qualeffo-41 | Habitual walking speed | 10-meter walking test | Physical fitness | Senior Fitness Test, Functional Reach, and Four Square Step Test |
|  |  |  |  |  |  | HRQOL | - |
|  |  |  |  |  |  | Fear of falling | Falls Efficacy Scale International |
|  |  |  |  |  |  | Physical activity levels | IPAQ-SF |
| Zhang et al., 2022 (18) | Participants who underwent HBRE | Participants who underwent received usual care | SF-36 | Physical function | + HGS  + FTSST  + TUGT  + BBS | Exercise self-efficacy | ESES |
|  |  |  |  | Muscle strength |  | Falling efficacy | FES |
|  |  |  |  | Balance ability |  | HRQOL | SF-36 |

BBS, Berg Balance Scale; BDI-II, Beck Depression Inventory II; CES-D, Center for Epidemiologic Studies Depression Scale; CG, Control group; CTX, C-terminal telopeptide; DS, Depressive Symptoms; DXA, dual-energy X-ray absorptiometry; ESES, Exercise Self-Efficacy Scale; FES, Falling Efficacy Scale; FTSST, Five-Time Sit-to-Stand Test; HBRE, Home-based resistance exercise program; HGS, Handgrip Strength; HRQOL, health-related quality of life; JOQOL, Japanese Osteoporosis Quality of Life Questionnaire; mini-OQLQ, mini-Osteoporosis Quality of Life Questionnaire; NHP, Nottingham Health Profile; OC, osteocalcin; OFDQ, Osteoporosis Functional Disability Questionnaire; OQLQ, Osteoporosis Quality of Life Questionnaire; QUALEFFO-41, Quality of Life Questionnaire of the European Foundation for Osteoporosis; SF-36, Short Form 36; SG, Study Group; SoC, Sense of Coherence; SOC-13, 13-item Sense of Coherence Scale; SRT, Sensitized Romberg Test; TLS, Timed Loaded Standing; TSS, Time Sit to Stand Test; TUGT, Timed Up and Go Test; WBV, Whole-body vibration

## Table S7. Risk of bias assessment for the included randomized controlled trials (RoB 2 tool)

| Author, year (reference) | Random sequence generation (Selection bias) | Allocation concealment (Selection bias) | Blinding of participants and personnel (Performance bias) | Blinding of outcome assessment (Detection bias) | Incomplete outcome data (Attrition bias) | Selective reporting (Reporting bias) | Other bias | Overall risk of bias |
| --- | --- | --- | --- | --- | --- | --- | --- | --- |
| Alp et al., 2009 (1) | Low (+) | Unclear (?) | High (–) | Low (+) | Low (+) | Low (+) | Unclear (?) | Moderate |
| Arnold et al., 2008 (2) | Low (+) | Unclear (?) | High (–) | Low (+) | Low (+) | Low (+) | Low (+) | Moderate |
| Carter et al., 2002 (3) | Low (+) | Unclear (?) | High (–) | Low (+) | Low (+) | Low (+) | Low (+) | Moderate |
| Cergel et al., 2019 (4) | Low (+) | Low (+) | High (–) | Low (+) | Low (+) | Low (+) | Low (+) | Moderate |
| Evstigneeva et al., 2016 (5) | Low (+) | Low (+) | High (–) | Low (+) | Low (+) | Low (+) | Low (+) | Moderate |
| Ferrara et al., 2019 (6) | Unclear (?) | Unclear (?) | High (–) | Unclear (?) | Low (+) | Low (+) | Low (+) | Moderate |
| Fu and Fan, 2021 (7) | Low (+) | Unclear (?) | High (–) | Unclear (?) | Low (+) | Low (+) | Low (+) | Moderate |
| Gibbs et al., 2020 (8) | Low (+) | Low (+) | High (–) | Low (+) | Low (+) | Low (+) | Low (+) | Moderate |
| Hongo et al., 2007 (9) | Low (+) | Low (+) | High (–) | Low (+) | Low (+) | Low (+) | Low (+) | Moderate |
| Kanemaru et al., 2010 (10) | Unclear (?) | Unclear (?) | High (–) | Low (+) | Low (+) | Low (+) | Low (+) | Moderate |
| Kekalainen et al., 2018 (11) | Low (+) | Low (+) | High (–) | Low (+) | Low (+) | Low (+) | Low (+) | Moderate |
| Khalili et al., 2017 (12) | Low (+) | Low (+) | High (–) | Low (+) | Low (+) | Low (+) | Low (+) | Moderate |
| Grahn Kronhed et al., 2009 (13) | Unclear (?) | Unclear (?) | High (–) | Unclear (?) | Low (+) | Low (+) | Low (+) | Moderate |
| Kucukcakir et al., 2013 (14) | Low (+) | Low (+) | High (–) | Low (+) | Low (+) | Low (+) | Low (+) | Moderate |
| Matthews et al., 2020 (15) | Low (+) | Low (+) | High (–) | Low (+) | Low (+) | Low (+) | Low (+) | Moderate |
| Sen et al., 2020 (16) | Low (+) | Unclear (?) | High (–) | Low (+) | Low (+) | Low (+) | Low (+) | Moderate |
| Stanghelle et al., 2020 (17) | Low (+) | Low (+) | High (–) | Low (+) | Low (+) | Low (+) | Low (+) | Moderate |
| Zhang et al., 2022 (18) | Low (+) | Low (+) | High (–) | Low (+) | Low (+) | Low (+) | Low (+) | Moderate |

## Table S8. Summary of GRADE assessment for each main outcome

| Outcome / Domain | No. of Studies | Risk of Bias | Inconsistency | Indirectness | Imprecision | Publication Bias | Overall Certainty |
| --- | --- | --- | --- | --- | --- | --- | --- |
| Overall HRQoL | 18 | Low | Serious | Not serious | Not serious | Possible | Moderate |
| Resistance training | 4 | Low | Not serious | Not serious | Not serious | Possible | Moderate |
| Multicomponent exercise | 5 | Low | Serious | Not serious | Serious | Possible | Low |
| Short-term (< 20 weeks) | 6 | Low | Not serious | Not serious | Not serious | Unlikely | Moderate |
| Medium-term (~6 months) | 3 | Some concerns | Serious | Not serious | Serious | Possible | Low |
| Long-term (~12 months) | 2 | Some concerns | Serious | Serious | Serious | Unclear | Very Low |
| Physical HRQoL domains | 15 | Low | Serious | Not serious | Not serious | Possible | Moderate |
| Mental HRQoL domains | 13 | Some concerns | Serious | Not serious | Serious | Possible | Low |

Participant numbers reflect those contributing to each pooled analysis and may differ from the total sample size across included trials.

# References

1. Alp A, Cansever S, Görgeç N, Yurtkuran M, Topsaç T. Effects of Tai Chi exercise on functional and lifequality assessments in senile osteoporosis. Turkiye Klin J Med Sci. 2009;29 (3):687-95.

2. Arnold CM, Busch AJ, Schachter CL, Harrison EL, Olszynski WP. A Randomized Clinical Trial of Aquatic versus Land Exercise to Improve Balance, Function, and Quality of Life in Older Women with Osteoporosis. Physiother Can. 2008;60(4):296-306. doi:10.3138/physio.60.4.296

3. Carter ND, Khan KM, McKay HA, Petit MA, Waterman C, Heinonen A, Janssen PA, Donaldson MG, Mallinson A, Riddell L, Kruse K, Prior JC, Flicker L. Community-based exercise program reduces risk factors for falls in 65- to 75-year-old women with osteoporosis: randomized controlled trial. CMAJ. 2002;167(9):997-1004.

4. Cergel Y, Topuz O, Alkan H, Sarsan A, Sabir Akkoyunlu N. The effects of short-term back extensor strength training in postmenopausal osteoporotic women with vertebral fractures: comparison of supervised and home exercise program. Arch Osteoporos. 2019;14(1):82. doi:10.1007/s11657-019-0632-z

5. Evstigneeva L, Lesnyak O, Bultink IE, Lems WF, Kozhemyakina E, Negodaeva E, Guselnikova G, Belkin A. Effect of twelve-month physical exercise program on patients with osteoporotic vertebral fractures: a randomized, controlled trial. Osteoporos Int. 2016;27(8):2515-24. doi:10.1007/s00198-016-3560-4

6. Ferrara PE, Salini S, Maggi L, Foti C, Maccauro G, Ronconi G. Evaluation of quality of life and static balance in postmenopausal osteoporosis women after Tai Chi Chuan practice: an observational randomized case control study. J Biol Regul Homeost Agents. 2019;33(2 Suppl. 1):163-9 XIX Congresso Nazionale S I C O O P Societa' Italiana Chirurghi Ortopedici Dell'ospedalita' Privata Accreditata. doi:10.54517/jbrha6738

7. Fu W, Fan J. Intervention effect of exercise rehabilitation therapy on patients with type 2 diabetic osteoporosis. Am J Transl Res. 2021;13(4):3400-8.

8. Gibbs JC, McArthur C, Wark JD, Thabane L, Scherer SC, Prasad S, Papaioannou A, Mittmann N, Laprade J, Kim S, Khan A, Kendler DL, Hill KD, Cheung AM, Bleakney R, Ashe MC, Adachi JD, Giangregorio LM. The Effects of Home Exercise in Older Women With Vertebral Fractures: A Pilot Randomized Controlled Trial. Phys Ther. 2020;100(4):662-76. doi:10.1093/ptj/pzz188

9. Hongo M, Itoi E, Sinaki M, Miyakoshi N, Shimada Y, Maekawa S, Okada K, Mizutani Y. Effect of low-intensity back exercise on quality of life and back extensor strength in patients with osteoporosis: a randomized controlled trial. Osteoporos Int. 2007;18(10):1389-95. doi:10.1007/s00198-007-0398-9

10. Kanemaru A, Arahata K, Ohta T, Katoh T, Tobimatsu H, Horiuchi T. The efficacy of home-based muscle training for the elderly osteoporotic women: the effects of daily muscle training on quality of life (QoL). Arch Gerontol Geriatr. 2010;51(2):169-72. doi:10.1016/j.archger.2009.10.003

11. Kekalainen T, Kokko K, Sipila S, Walker S. Effects of a 9-month resistance training intervention on quality of life, sense of coherence, and depressive symptoms in older adults: randomized controlled trial. Qual Life Res. 2018;27(2):455-65. doi:10.1007/s11136-017-1733-z

12. Khalili A, Almasi MH, Raeissadat SA, Sedighipour L, Salek Zamani Y, Zohoor MRO. Long-term effects of back extensor strengthening exercises on quality of life in women with osteoporosis. J Women Aging. 2017;29(6):505-14. doi:10.1080/08952841.2016.1223968

13. Grahn Kronhed A-C, Hallberg I, Ödkvist L, Möller M. Effect of training on health-related quality of life, pain and falls in osteoporotic women. Adv Physiother. 2009;11(3):154-65. doi:10.1080/14038190902896659

14. Kucukcakir N, Altan L, Korkmaz N. Effects of Pilates exercises on pain, functional status and quality of life in women with postmenopausal osteoporosis. J Bodyw Mov Ther. 2013;17(2):204-11. doi:10.1016/j.jbmt.2012.07.003

15. Matthews J, Torres SJ, Milte CM, Hopkins I, Kukuljan S, Nowson CA, Daly RM. Effects of a multicomponent exercise program combined with calcium-vitamin D(3)-enriched milk on health-related quality of life and depressive symptoms in older men: secondary analysis of a randomized controlled trial. Eur J Nutr. 2020;59(3):1081-91. doi:10.1007/s00394-019-01969-8

16. Sen EI, Esmaeilzadeh S, Eskiyurt N. Effects of whole-body vibration and high impact exercises on the bone metabolism and functional mobility in postmenopausal women. J Bone Miner Metab. 2020;38(3):392-404. doi:10.1007/s00774-019-01072-2

17. Stanghelle B, Bentzen H, Giangregorio L, Pripp AH, Skelton DA, Bergland A. Effects of a resistance and balance exercise programme on physical fitness, health-related quality of life and fear of falling in older women with osteoporosis and vertebral fracture: a randomized controlled trial. Osteoporos Int. 2020;31(6):1069-78. doi:10.1007/s00198-019-05256-4

18. Zhang F, Wang Z, Su H, Zhao H, Lu W, Zhou W, Zhang H. Effect of a home-based resistance exercise program in elderly participants with osteoporosis: a randomized controlled trial. Osteoporos Int. 2022;33(9):1937-47. doi:10.1007/s00198-022-06456-1
